# Plastic Products Leach Chemicals That Induce *In Vitro* Toxicity under Realistic Use Conditions

**DOI:** 10.1021/acs.est.1c01103

**Published:** 2021-08-17

**Authors:** Lisa Zimmermann, Zdenka Bartosova, Katharina Braun, Jörg Oehlmann, Carolin Völker, Martin Wagner

**Affiliations:** † Department Aquatic Ecotoxicology, Goethe University Frankfurt am Main, Max-von-Laue-Str. 13, 60438 Frankfurt, Germany; ‡ Department of Biology, Norwegian University of Science and Technology (NTNU), Høgskoleringen 5, 7491 Trondheim, Norway; § Institute for Social-Ecological Research, Hamburger Allee 45, 60486 Frankfurt am Main, Germany

**Keywords:** food contact materials, polymers, additives, exposure, exposome, migration, bioassays, nontarget

## Abstract

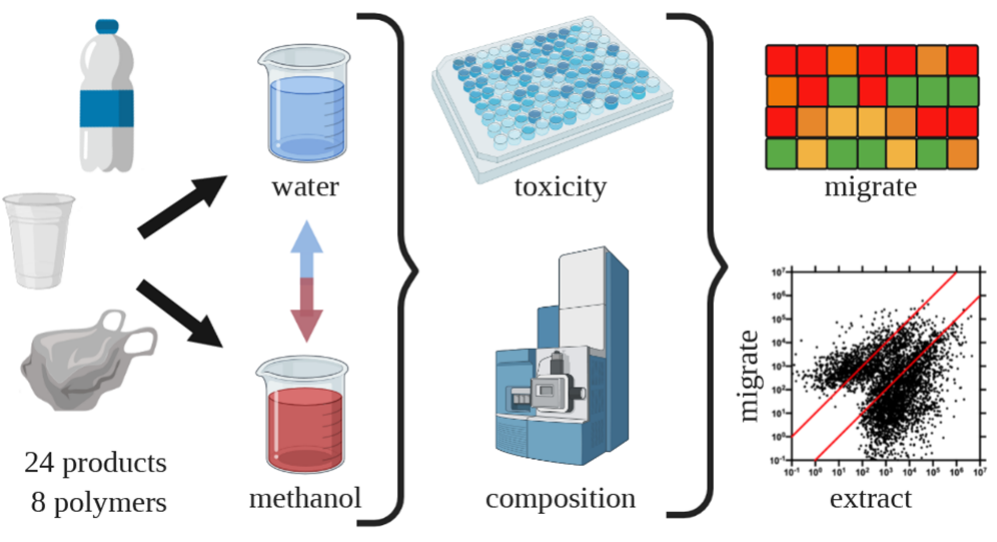

Plastic products contain complex mixtures of extractable chemicals that can be toxic. However, humans and wildlife will only be exposed to plastic chemicals that are released under realistic conditions. Thus, we investigated the toxicological and chemical profiles leaching into water from 24 everyday plastic products covering eight polymer types. We performed migration experiments over 10 days at 40 °C and analyzed the migrates using four *in vitro* bioassays and nontarget high-resolution mass spectrometry (UPLC-QTOF-MS^E^). All migrates induced baseline toxicity, 22 an oxidative stress response, 13 antiandrogenicity, and one estrogenicity. Overall, between 17 and 8681 relevant chemical features were present in the migrates. In other words, between 1 and 88% of the plastic chemicals associated with one product were migrating. Further, we tentatively identified ∼8% of all detected features implying that most plastic chemicals remain unknown. While low-density polyethylene, polyvinyl chloride, and polyurethane induced most toxicological endpoints, a generalization for other materials is not possible. Our results demonstrate that plastic products readily leach many more chemicals than previously known, some of which are toxic *in vitro*. This highlights that humans are exposed to many more plastic chemicals than currently considered in public health science and policies.

## Abstract 2

Here, we demonstrate that hundreds to thousands of chemicals migrate into water from plastic products and that these leachates induce toxicity *in vitro*. This highlights that plastic chemicals leach under realistic conditions and, thus, can be a relevant contributor to the human exposome.

## 1 Introduction

Individual plastic chemicals, such as bisphenol A and phthalates, have received much scientific and public attention. However, plastics are not composed of single compounds but contain a wide variety of chemicals:^1^ more than 4000 chemicals have been associated with plastic packaging alone.^2^ These include starting substances such as monomers, oligomers, and polymers as well as additives, including plasticizers, antioxidants, heat stabilizers, and pigments. In addition, plastics contain an unknown number of non-intentionally added substances (NIAS), that is, impurities of the starting substances and additives as well as intermediates, and reaction and breakdown products formed during processing.^3^ The total number of plastic chemicals, consisting of intentionally and non-intentionally added substances, is unknown as is their mixture toxicity. Thus, we extracted everyday plastics with methanol in our previous study and demonstrated that they contain complex chemical mixtures that induce *in vitro* toxicity.^4^

Since most plastic chemicals are not covalently bound to the polymer matrix, they can leach into the packaged goods in case of packaging. In the context of human health, such chemical migration is especially relevant for food contact materials (FCMs) as compounds leaching into foodstuff will become available for human exposure. Plastic chemicals can also leach into natural environments from littering, resulting in the exposure of wildlife. Previous studies have demonstrated that the chemicals migrating into aqueous media include organic compounds and metals,^5^ phenols and phthalates,^6,7^ as well as known estrogenic chemicals.^8^ Emerging research using nontarget analysis has expanded this spectrum greatly, especially with respect to NIAS.^9−12^ However, concerns have been raised regarding the lack of hazard information for chemicals known to be present in FCMs, including plastics, as well as the challenge of unknown compounds migrating from such materials.^13^

One approach to tackle the chemical complexity of plastics, including the large number of unknown chemicals and mixture effects, is whole migrate toxicity testing.^14^ Indeed, *in vitro* bioassays have been applied already to determine the overall toxicity of the chemical mixtures leaching from plastics.^15^ Here, plastic migrates induced unspecific effects in *Aliivibrio fischeri*(16) and *Photobacterium phosphoreum*,^17^ cytotoxicity, and endocrine activity.^8,18,19^ However, a comprehensive comparison of the extractable chemicals present in plastics and the compounds leaching under more realistic conditions including their toxicity is missing.

Thus, we selected 24 plastic products covering eight polymer types, performed migration experiments with water, and analyzed these migrates for baseline toxicity, oxidative stress induction, and endocrine activity. Subsequently, we compared the *in vitro* effects with those induced by methanolic extracts of the same samples.^4^ In addition, we performed nontarget high-resolution mass spectrometry (UPLC-QTOF-MS^E^) to characterize and compare the extractable and leachable chemicals. Accordingly, our results shed light on the fraction of plastic chemicals and their toxicity available for human and wildlife exposure.

## 2 Material and Methods

### 2.1 Sample Selection

We acquired 24 commonly used plastic products available on the German market (exception: PVC 1 from the Chinese market, Table 1). These covered eight polymer types (high-density and low-density polyethylene, HDPE and LDPE; polystyrene, PS; polypropylene, PP; polyethylene terephthalate, PET; polyvinyl chloride, PVC; polyurethane, PUR; and polylactic acid, PLA). These samples induced *in vitro* toxicity in our previous study when extracted with methanol by sonication for 1 h at room temperature (HDPE 1 corresponds to HDPE 3, PP 1 to PP 2, PP 2 to PP 3, and PET 1 to PET 3 of our previous study).^4^ Besides all active products (but PP 5, as it was removed from the assortment), we included LDPE 3 as a representative of nontoxic products. Half of the 24 products were FCMs. We purchased the products in local retailer stores and confirmed their polymer types using Fourier-transform infrared spectroscopy (FTIR, PerkinElmer, Spectrum Two, Waltham, Massachusetts) in our previous study. The spectra of the samples are available under DOI: 10.5281/zenodo.3263830.

**Table 1:** Plastic Products Analyzed in this Study

sample	plastic product	FCM^a^
HDPE 1	bin liners	
LDPE 1	lemon juice bottle	+
LDPE 2	plastic wrap	+
LDPE 3	freezer bag	+
LDPE 4	hair conditioner bottle	
PS 1	yogurt cup	+
PS 2	fruit tray	+
PS 3	vegetable tray	+
PS 4	plastic cup	+
PP 1	yogurt cup	+
PP 2	gummi candy packaging	+
PET 1	oven bag	+
PVC 1	plastic wrap	+
PVC 2	placemat	
PVC 3	pond liner	
PVC 4	floor covering	
PUR 1	scouring pad	
PUR 2	kids bath sponge	
PUR 3	acoustic foam	
PUR 4	shower slippers	
PLA 1	yogurt cup	+
PLA 2	vegetable tray	+
PLA 3	shampoo bottle	
PLA 4	coffee cup lid	+

a FCM: Food contact material.

### 2.2 Migration Experiment

To avoid sample contamination, we used glass or poly(tetrafluoroethylene) (PTFE) consumables whenever feasible, rinsed all materials twice with acetone (pico-grade, LGC Standards), and annealed glass items at 200 °C for ≥3 h. Additionally, we conducted the sample preparation and the bioassays under a laminar flow hood. For sample preparation, the content was removed from the packaging samples and the products were rinsed thoroughly with ultrapure water until all residues were removed. All samples were cut into 0.5–1.5 × 2 cm pieces. The surface areas of the products varied due to the different thicknesses of the samples. Therefore, we decided to cut foamy products to a thickness of 0.5 cm as well as to extract the same masses instead of surface areas.

Based on the results of an initial experiment (see the Supporting Information for details), migration conditions were set to 10 days at 40 °C in the dark which corresponds to the migration testing conditions laid out in the EU regulation for plastic FCMs.^20^ 60.8 g were leached in 1520 mL of ultrapure water (exception PET 1: 30.8 g in 760 mL), corresponding to 40 mg plastic mL^–1^ water. After 10 days, the solution was filtered through porcelain funnels into new 2 L glass bottles. Foamy samples were additionally squeezed using syringes to recover most of the water. The recovered volume of ultrapure water was determined, 20 mL was transferred into new brown glass vials, and stored at 8 °C (aqueous migrates). The remaining sample was extracted using solid-phase extraction (SPE; migrates). To contextualize the bioassay results, we use plastic equivalents such that “1 mg plastic” represents the toxicity migrating from 1 mg plastic per well. One well contained 150 μL volume in the Microtox assay, 100 μL volume in the AREc32 assay, and 120 μL volume in the Yeast Estrogen Screen (YES) and the Yeast Antiandrogen Screen (YAAS).

### 2.3 Solid-Phase Extraction

We used C18-silica gel cartridges (TELOS C18(EC)/ENV, 700 mg, 6 mL, 697-70M-006Z, Kinesis, Wertheim, Germany) to extract the aqueous samples. SPE columns were sequentially conditioned with 2 mL *n*-heptane (Carl Roth, CAS: 142-82-5, purity ≥ 99.9%), followed by 2 mL acetone (Carl Roth, CAS: 67-64-1, ≥99.9%), 6 mL methanol (Carl Roth, CAS: 67-56-1, ≥99.95%), and 8 mL ultrapure water by gravity. The pH of the aqueous samples was adjusted to 2.5 using 3.5 M sulfuric acid (VWR, CAS: 7664-93-9, 96%) before loading on the columns with a constant vacuum flow of approximately 2–5 mL min^–1^. The cartridges were dried under a gentle stream of nitrogen and eluted with 5 mL acetone followed by 5 mL methanol. The combined extracts were evaporated to dryness under nitrogen and redissolved in approximately 150 μL of dimethyl sulfoxide (DMSO, Carl Roth, CAS: 67-68-5, ≥99.9%). The volume of DMSO was adjusted to the volume of each sample to generate extracts that are 10 000 times concentrated and equivalent to 400 mg plastic μL^–1^ DMSO. These migrates were stored in glass vials with PTFE caps at −20 °C prior to analysis.

Six procedural blanks (PB 1–6, two per run) consisting of glass bottles not containing any sample but only ultrapure water and three SPE blanks (SPE 1–3, one per run) consisting of 1.5 L of ultrapure water directly applied to SPE were treated identically (using the same solvent batches) to control for potential contamination.

### 2.4 Bioassays

All bioassays were conducted in 96-well microtiter plates with negative controls, solvent controls (DMSO for migrates only), PB 1–6, and SPE 1–3. Aqueous migrates, solvent controls, and blanks in ultrapure water were diluted 1.4-fold (baseline toxicity) and 1.6-fold (endocrine activity). Migrates in DMSO were diluted 100-fold (baseline toxicity), 200-fold (oxidative stress response), or 480-fold (endocrine activity) with the medium, resulting in a maximum final solvent concentration of 1, 0.5, or 0.2% (v/v), respectively. Throughout the main experiments, none of the blanks induced toxicity (Figures S1 and S2). Thus, there was no contamination during migration, extraction, and analysis, and pooled blanks (control, C) are presented in bioassay results (Figures S4–S8).

#### 2.4.1 Baseline Toxicity

The Microtox assay with the bioluminescent bacterium *Allivibrio fischeri* was performed according to an ISO guideline^21^ as described previously^4^ with minor modification for testing aqueous migrates. These were adjusted to a conductivity of 25–45 mS cm^–1^ by the addition of sodium chloride. Subsequently, *A. fischeri* suspension (50 μL) was added to 125 μL of the aqueous migrate. Negative and positive controls (3,5-dichlorophenol, Table S3 and Figure S3) and migrates were analyzed in 1:2 serial dilutions corresponding to concentrations of 39.1 μg to 5.0 mg and 18.31 μg to 600 mg plastic well^–1^ (PVC: 71.53 ng to 600 mg) for aqueous migrates and migrates, respectively. Results from three to six independent experiments (dots in the graph), each with two technical replicates, were expressed as effect concentration (EC_20_, EC_50_ ± standard error of the mean (SEM), mass of plastic well^–1^ inducing a 20, 50% luminescence inhibition) and mean effect size ± SEM (luminescence inhibition induced by 22.5 mg plastic well^–1^) if 20 or 50% inhibition was reached, respectively, in *n* ≥ 1. In case an EC_20_ or EC_50_ could not be derived, we used an EC of 6.25 mg plastic well^–1^ for aqueous migrates and 750 mg plastic well^–1^ for migrates to visualize the data, indicating that the EC is larger than the highest analyzed concentration (HAC).

#### 2.4.2 Oxidative Stress Response

We used the AREc32 assay to investigate the induction of an oxidative stress response in the Nrf2/ARE pathway.^22^ The AREc32 assay and the determination of cell viability were performed as described elsewhere.^23^ We analyzed eight concentrations of the migrates in serial dilutions (1:2, 1.56–200 mg plastic well^–1^) and the reference compound *tert*-butylhydroquinone (*t*-BHT, Table S3 and Figure S3). Each sample was analyzed in three independent experiments (dots in the graph) with duplicates each. We excluded concentrations that were cytotoxic in the respective experiment and replicate before deriving induction ratios (IRs) as well as the effect concentration producing an IR of 2 over the control (EC_IR2_). In case an EC_IR2_ could not be derived, we used an EC_IR2_ of 250 mg plastic well^−1^ to visualize the data, indicating that the EC_IR2_ is larger than the HAC.

#### 2.4.3 Endocrine Activity

We used yeast-based reporter-gene assays to investigate the induction of agonistic activity at the human estrogen receptor α (hERα)^24^ and antagonistic activity at the human androgen receptor (hAR).^25^ The YES and the YAAS with the reference compounds, 17β-estradiol and flutamide (Table S3 and Figure S3), respectively, were performed as previously described with minor modifications.^4^ Samples were analyzed in concentrations of 3.0 mg (aqueous migrates) or 0.2–100 mg plastic well^–1^ (migrates) and in two to four independent experiments with eight replicates, each. Cytotoxic migrates were analyzed in 1:2 serial dilutions down to 9.9 ng plastic well^–1^ (PLA 3) in the YES and 0.02 ng plastic (PP 3), 9.90 ng plastic (PLA 3), and 0.38 μg plastic well^–1^ (PLA 4) in the YAAS assay. The limit of detection (LOD) of each experiment was calculated as three times the standard deviation (SD) of pooled negative and solvent controls. Mean effects > LOD were considered significant. Plastic equivalents inducing 20% cytotoxicity (EC_20_) and 50% relative endocrine activity (EC_50_, calculated if *n* ≥ 1 had a relative activity > 50%) are reported. In case an EC_50_ could not be derived, we used an EC_50_ of 3.75 mg for aqueous migrates and 125 mg plastic well^–1^ for migrates, indicating that the EC_50_ is larger than the HAC. To ensure comparability, only those experiments were considered in which the concentration–response relationship of the reference compound had an *r*^2^ > 0.9, a minimal relative luminescence unit <5000, a maximal luminescence unit >50 000, and an EC_50_ next to 8 × 10^–11^ mol L^–1^ 17β-estradiol (YES) or 1.5 × 10^–5^ mol L^–1^ flutamide (YAAS, Table S3).

#### 2.4.4 Analysis of Bioassay Data

**Figure 1:**
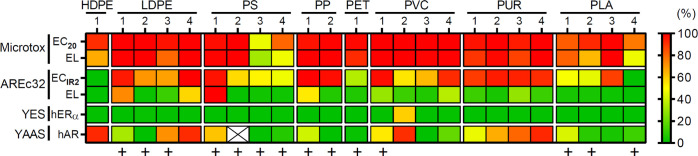
*In vitro* toxicity of chemicals migrating from plastic consumer products. Baseline toxicity (Microtox) and oxidative stress response (AREc32) are presented as effect concentrations inducing 20% baseline toxicity (EC_20_) or an induction ratio of 2 (EC_IR2_) as well as effect levels (EL) at the highest analyzed noncytotoxic concentration. Estrogenic (YES) and antiandrogenic activities (YAAS) are shown as relative (%) activation of the human estrogen receptor α (hERα) and inhibition of the androgen receptor (hAR). Note: x, all analyzed concentrations were cytotoxic; +, food contact materials.

**Figure 2:**
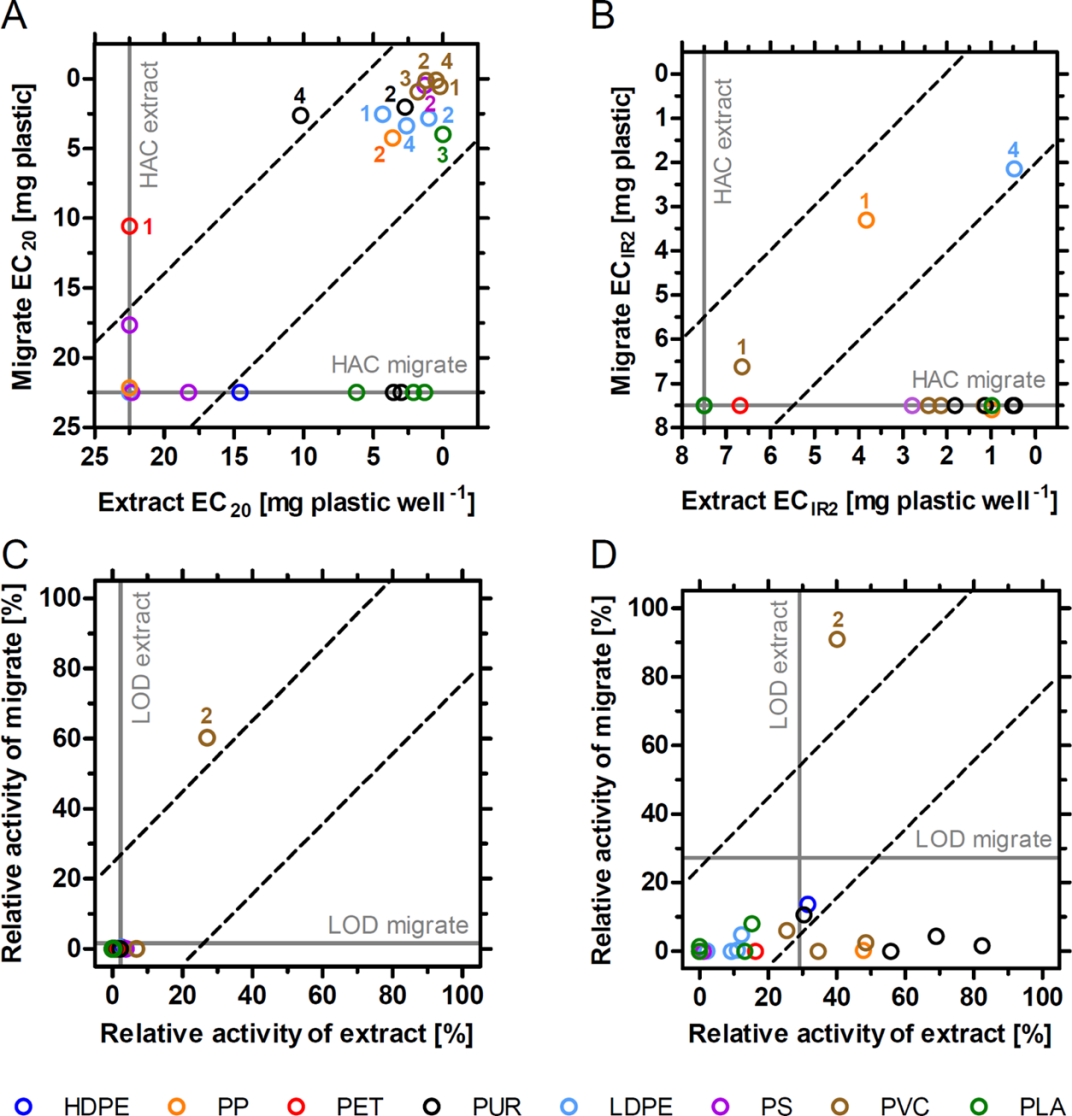
Comparison of *in vitro* toxicity present in plastics (extracts) and leaching from plastics (migrates). For baseline toxicity (A) and oxidative stress induction (B), effect concentrations (EC_20_, EC_IR2_) up to the highest analyzed concentration (HAC) measured for both, migrates and extracts, were plotted. Relative estrogenic (C) and antiandrogenic (D) activities were correlated at 3.75 mg plastic well^–1^. Sample numbers are given if migrate and extract shared similar toxicity (between the dotted lines), or if the migrate was more toxic (above the upper dotted line). Note: LOD, limit of detection.

We used GraphPad Prism 5 and 8 (GraphPad Software, San Diego, CA) for nonlinear regressions (four-parameter logistic models) and statistical analyses. To present toxicities of plastic migrates in a heat map (Figure 1), *in vitro* data were plotted as a gradient from 0 (green) to 100% (red) toxicity. The endocrine activities were used as such. Effects in the Microtox and AREc32 assay were normalized to the lowest and highest effect observed for the respective endpoint. For AREc32 effect levels (ELs) and for endocrine activities the highest noncytotoxic concentrations (Tables S5 and S6) were used. For the comparison of extracts^4^ and migrates (Figure 2), ECs were set to the HAC and endocrine activities to zero in case the sample did not induce an effect. If cytotoxicity occurred, the highest concentration that was nontoxic for both, extract and migrate, was compared (antiandrogenic activity: PVC 2, 0.78 mg; estrogenic activity: PVC 2 and PLA 1, 0.94; PS 2, 0.47; PLA 3, 0.03 mg plastic well^–1^).

### 2.5 Chemical Analysis

The nontarget screening was conducted using an ultrahigh-performance liquid chromatograph AQUITY I-Class UPLC coupled to a hybrid quadrupole orthogonal time-of-flight mass spectrometer SYNAPT G2-S HDMS (both Waters, Milford, MA). The UPLC system was equipped with a binary pump, an online vacuum degasser, an autosampler, and a thermostated column compartment. The separation was carried out on an Acquity UPLC BEH C18 column (130 Å, 1.7 μm, 2.1 × 150 mm^2^) equipped with a guard column C18 (both Waters), with mobile phases (A) H_2_O and (B) methanol, both with 0.1% formic acid. The gradient of B was set as follows: 0 min, 20%; 0.5 min, 20%; 4.5 min, 60% (6); 35.5 min, 100% (6); 38.5 min, 100%; 39.5 min, 20% (6); and 41.5 min, 20%. The column temperature was maintained at 40 °C, the flow rate was set to 0.2 mL min^–1^, and the injection volume was 2 μL.

The mass spectrometer was equipped with an ESI source operated in a positive mode. The MS detection conditions were set as follows: capillary voltage, 2.5 kV; cone voltage, 30; source offset voltage, 60 V; source temperature, 120 °C; desolvation temperature, 350 °C; desolvation gas flow, 800 L h^–1^; cone gas flow, 100 L h^–1^; collision gas flow, 0.15 mL min^–1^; and nebulizer gas pressure, 6 bar. The mass spectrometer was operated in data-independent MS^E^ acquisition mode and the high collision energy was ramped from 15 to 45 eV. Data were acquired from 2 to 35 min over the mass range of 50–1200 Da, and the resolution of the mass spectrometer was 20 000.

Prior to analysis, the migrates and the extracts of our previous study^4^ stored in DMSO in glass vials were diluted in 1:1 methanol/water (v/v) by 62.5- and 1667-fold, respectively, to yield a concentration of 0.24 mg plastic μL^–1^. Each sample was analyzed once. Quality controls (QCs) were prepared by pooling aliquots of each individual sample: one QC was prepared for the extracts and another one for the migrates. LC blanks (1:1 methanol/water) and QCs were injected regularly after 10 sample injections to check for contamination and monitor the performance of the instrument. The mass spectral data of all samples can be accessed under DOI: 10.18710/COLBSF.

#### 2.5.1 Data Analysis and Compound Identification

We used Progenesis QI (version 2.3, Nonlinear Dynamics) to analyze the UPLC-QTOF-MS/MS data. In brief, we imported the raw data files of PBs (six for the migrates and two for the extracts) and of the extracts and migrates of each plastic sample individually. The lock-mass correction with leucine enkephalin was done online. We enabled the search for common adducts (M+H, M+H−H_2_O, M+Na, M+CH_3_OH+H, M+K, 2M+H, 2M+Na, 2M+K), automatically aligned the retention times of all runs, and performed the peak picking (automatic sensitivity, 0.02 min minimum peak width, retention times <2 min excluded, fragment sensitivity of 0.2% of the base peak).

We exported the resulting feature list to Microsoft Excel for Mac (version 16.35) and compared the maximum raw abundance of each feature in the PBs (*n* = 8) to the raw abundance of the same feature in the extract or the migrate of the respective sample. We filtered for features that were not present in PBs, but in the sample, or had a 10-fold higher abundance in the sample than in the PBs. The resulting feature list represents all chemicals detected in either the extract, the migrate, or both. We determined the ratio of the raw abundance of each feature in the migrate and the extract to determine how many features did not migrate (ratio < 0.1), migrated (ratio > 0.1), or were newly formed in water (not present in extracts). The migration cut-off is based on the assumption that if a compound has less than 10% abundance in the migrate compared to the extract, migration would be low. While this represents a pragmatic approach, the concentration of chemicals classified as “not migrating” might nonetheless be significant.

We tentatively identified all features detected in the samples using the Metascope algorithm in Progenesis QI for comparison with empirical spectra from MassBank and *in silico* fragmentation. In brief, we downloaded the MassBank spectral library containing 14 788 unique compounds (release version 2021.03) in the NIST msp format from https://github.com/MassBank/MassBank-data/releases/tag/2021.03. For *in silico* fragmentation, we constructed three databases (see the Supporting Information for details) covering the chemicals present in plastic packaging (CPPdb, 2680 compounds, including the compounds on the positive list of the European plastic regulation 10/2011),^2^ the chemicals registered under the REACH regulation in 2020 (ECHAdb, 7092 compounds),^26^ and the chemicals (pre)registered under REACH in 2017 as provided by the NORMAN Suspect List Exchange (NORMANdb, 65 738 compounds).^27^ These databases were queried individually for each sample with a precursor tolerance of 5 ppm and a fragment tolerance of 10 ppm. The results of the tentative identification were filtered for hits with a score > 40 (based on fragmentation, mass, and isotope similarity, max. 60). If a feature had multiple identifications with a score > 40, the one with the highest score was picked. The results of the identification with the four databases were combined and duplicates were removed retaining the identification with the highest score per feature.

## 3 Results and Discussion

In our previous study, we demonstrated that consumer plastics contain extractable chemicals inducing *in vitro* toxicity.^4^ Since exposure only occurs if these extractable compounds also leach under realistic conditions, we performed migration experiments with water using the conditions set out by the European Union regulation on FCMs.^20^ It is assumed that the toxicity of the migrate can serve as an indicator for the chemical toxicity readily released from the plastic product in conditions commonly encountered during use or after disposal (*e.g*., migration into packed foodstuff, leaching in aquatic environments).

### 3.1 Plastic Products Leach Toxicity

All plastic products we investigated leached chemicals triggering *in vitro* toxicity (Figure 1).

#### 3.1.1 Baseline Toxicity

Each sample induced baseline toxicity with the PVC migrates (1, 2, and 3) being most potent (EC_50_ < 5 mg plastic well^–1^, Table S4 and Figure S4). The widespread induction of baseline toxicity is in accordance with previous research^28^ and shows that migrating plastic chemicals trigger unspecific toxicity. The fact that all samples were active in the Microtox assays is probably related to our sample selection (based on the toxicity of the extracts) and the fact that a broad range of compounds causes baseline toxicity.^29^

#### 3.1.2 Oxidative Stress Response

In addition, all samples except HDPE 1 and PLA 4 activated the Nrf2-ARE-regulated oxidative stress response (Table S5 and Figure S5). Here, LDPE 4 was most potent (EC_IR2_ = 2.15 mg plastic well^–1^) and PS 1 had the highest effect level (IR = 80). A widespread release of chemicals inducing an oxidative stress response from plastic products has not been reported in the literature, so far. However, the leachates of UV-weathered PE, PET, PP, and PS microplastics were more active in the AREc32 assays than dark controls, indicating that photodegradation products resulting from intense UV A and B irradiation contribute to the oxidative stress induction.^30^

#### 3.1.3 Endocrine Activity

PVC 2 was the only sample that leached estrogen receptor agonists above the LOD (2.3%) with a relative activity of 59.4% at 1.56 mg plastic well^–1^ and an EC_50_ of 0.27 mg plastic well^–1^ (Table S6 and Figure S6A). The chemicals migrating from PVC 2 also induced the strongest antiandrogenic effects (EC_50_ = 0.28 mg plastic well^–1^). In total, 13 samples inhibited the androgen receptor above the LOD (27.3%, Table S6 and Figure S7A). PUR 4, HDPE 1, LDPE 4, and PVC 2 had an antiandrogenicity > 90% at 100 mg plastic well^–1^. This is in line with a number of studies that demonstrate the leaching of estrogenic or antiandrogenic compounds from multiple types of products and polymers.^8,19,31−35^ As an example, Berger et al.^31^ used the same assays but even lower temperatures (22 °C) and a shorter time period (24 h) for migration and reported that endocrine activity was triggered by chemicals migrating from 3.4 mg of plastic baby teethers. This conforms to our results that plastic masses in the lower milligram range leach chemical mixtures that can induce endocrine effects (*e.g*., migrates from <2 mg PVC 2 induced estrogenicity and antiandrogenicity, Table S6). Interestingly, our results show that the migrates’ antiandrogenicity was more pronounced and potent than their estrogenicity. This has been reported before for PP, PE, and PS FCMs.^19^ Importantly, the hypothesis that a stronger antiandrogenicity might be specific to yeast-based reporter-gene assays needs to be verified in future research.

We assessed the toxicity of migrates up to relatively high concentrations, covering the maximum equivalent of chemicals migrating from 100 mg (endocrine activity), 200 mg (oxidative stress), and 600 mg plastic well^–1^ (baseline toxicity). Nonetheless, many samples were very potent (EC_20_s well below 10 mg plastic well^–1^) and induced toxicity at low concentrations. As an example, the chemicals migrating from <0.3 mg of one PVC product, a material widely used in drinking water pipes in the EU and US and occasionally in cling films, induced 50% estrogenicity and antiandrogenicity (PVC 2, Figures S6 and S7). Taking into account that the mass of plastic products we use on a daily basis is much higher than in the milligram range, our results imply that human exposure to the chemicals inducing those effects is not negligible. In that context, it is important to emphasize that, based on our results, we cannot draw conclusions on human health impacts. This is because the actual exposure levels (*i.e*., in humans) and the *in vivo* toxicity of the mixture of chemicals leaching from plastics remain to be determined. Furthermore, we applied the official standard migration conditions for the testing of plastic FCMs (10 days at 40 °C) that have been set by the European Commission.^20^ While this is a regulatory accepted procedure mimicking the migration of chemicals from food packaging into foodstuff, it remains unclear how well these conditions reflect all the scenarios of plastic use. For instance, the migration of lipophilic compounds into fatty food is not well reflected when using water as food simulants. Besides the properties of the packaged good, several other factors influence the leaching of chemicals, including contact time, temperature, and area as well as the characteristics of the plastic product (*e.g*., thickness, polymeric structure, chemical properties).^36,37^ As a consequence, chemical migration and, thus, exposure levels, will change with the respective condition.

### 3.2 Toxicological Signature is Product-Specific

A comparison of the toxicological signatures of the migrates highlights that the toxicity migrating from plastics is specific to the product rather than the polymer type. Consistent with our previous results,^4^ the compounds migrating from PVC and PUR samples were very toxic. For instance, PVC 2 affected all endpoints with high potency. Eleven samples induced toxicity on three out of the four endpoints. These include all PUR migrates, three out of four LDPE migrates, and at least one sample of every other polymer type (exception: HDPE, PET with only one tested product). However, the levels of toxicity varied within all polymer type categories. As an example, the toxicity migrating from PS 3 and PLA 4 was much lower than the one observed for other samples made of the same polymers. This supports our notion that the chemical safety of plastic products cannot be generalized based on their polymer type.

Safety is of particular importance for products with food contact. Thus, we compared the toxicity of products intended for food contact (12 FCMs) with those not intended for food contact (12 non-FCMs, Table 1). Interestingly, both groups had a comparable potential to induce baseline toxicity and oxidative stress (Figure 1). More non-FCMs than FCMs induced antiandrogenicity but individual FCMs also released antiandrogenic compounds (*e.g*., LDPE 3, PS 1, PVC 1). This underpins concerns over the adequacy of the traditional approach of assessing the safety of FCMs that prescribes to assess the migration of starting substances.^20^ Concurrently, our results support the idea that whole migrate toxicity testing of the marketed products is a more appropriate approach to cover all plastic chemicals leaching from the final product.^13^

### 3.3 *In Vitro* Toxicity of Migrates and Extracts is Not Identical

To investigate whether the toxicity present in plastics leaches into water, we compared the effects of methanolic extracts and migrates using identical concentration ranges (Figure 2 and Table S7). For the former, we used the data from our previous study that was generated using the same samples and bioassays.^4^ The chemicals present in and leaching from eleven products induced a similar, high baseline toxicity (Figure 2A), including all PVC, three out of four LDPE as well as one PP, PS, and PLA products, each. Three PLA and two PUR products contained chemicals that inhibited bioluminescence but these did not migrate into water. In contrast, two products (PP 1, PUR 4) leached higher baseline toxicity in water than in methanol.

The chemicals activating an oxidative stress response were more readily extractable than leachable (Figure 2B). Here, the extracts and the migrates of LDPE 4, PP 1, and PVC 1 induced a similar toxicity. Ten other extracts activated this pathway with high efficiency but related migrates did not induce oxidative stress. With regard to endocrine effects, the estrogenicity detected in the extract of PVC 2 readily migrated into water (Figure 2C). Here, the estrogenicity of the migrate (60.3% at 3.75 mg well^–1^) was stronger than that of the extract (27.1%). The picture was similar for the antiandrogenicity of this sample (migrate: 90.9% *vs* extract: 40.1%, Figure 2D). Eight other products contained antiandrogenic chemicals > LOD_extract_ (29.2%) that did not leach into water.

As expected, these results show that not all *in vitro* toxicity detected in plastic extracts is migrating into water. This may be due to the fact that not all extractable chemicals are leaching and/or that the concentration of the leachable chemicals is lower than that of the extractable ones. Interestingly, chemicals inducing baseline toxicity had a higher migration potential than those triggering oxidative stress or antiandrogenic activity. Again, this might be related to the large number of compounds triggering baseline toxicity. In the case of migrate samples that induced a higher toxicity than that of their extract counterpart (PET 1, PUR 4), the causative compounds may dissolve better in water than in methanol. In addition, degradation products of the leaching compounds (*e.g*., by hydrolysis) might add to the toxicity. Both might also be true for the chemicals inducing endocrine activity in the migrate of PVC 2.

### 3.4 SPE Extracts the Toxicity from Aqueous Migrates but Needs Improvement

To assess the efficiency of the SPE to extract toxicity from the migrates, we also assessed the baseline toxicity (Figure S8) as well as the estrogenic (Figure S6B) and antiandrogenic activity (Figure S7B) of aqueous migrates (without SPE). When comparing the concentration–response relationships for baseline toxicity with migrates (with SPE), both sample types induced rather similar effects (Figure S9). However, for some samples (PS 1, PET 1, PLA 3), the baseline toxicity was higher in the aqueous migrates than in the extracted migrates (Table S8). With regard to the antiandrogenic activity, the aqueous migrate of one sample (PP 1, Table S8) induced an effect, whereas the corresponding migrate produced via SPE did not. This indicates that the compounds inducing toxicity were not recovered completely by the SPE method, similar to what has been observed for drinking water and wastewater.^38,39^ Accordingly, the sample preparation of aqueous media used for migration testing must be optimized to recover the maximum *in vitro* effects for future whole migrate toxicity testing.

### 3.5 Several Thousand Chemicals Migrate from Plastics

We detected between 278 (PS 3) and 15 815 (PUR 3) unique chemical features in the extracts and migrates of the 24 plastic products altogether (Table 2). Out of these, 75−3048 features were only detected in the extracts, that is, they were not migrating from the plastic products. Between 150 and 6307 features (low migration) were present in the extracts and migrates with at least 10-fold higher abundance in the former compared to the latter (ratio of <0.1). Thus, we classified these features as having a minor migration potential. In contrast, 14 (PS 4) to 8522 features (PUR 3) were readily leachable, that is, they were detected in the migrate with an abundance of at least 10% compared to the respective extract (ratio of >0.1). In addition, up to 611 features were only detected in migrates but not in extracts. This implies that these have been newly formed in water or are not extractable with methanol. In total, we found that between 17 (PS 4) and 8681 (PUR 3) features were either readily migrating from the plastic products or newly formed in the migrates. In half of the migrates, we detected more than 2000 chemical features. Thus, and in contrast to other studies using nontarget chemical analysis,^40,41^ we show that many more chemicals are migrating from plastic products than previously known. Importantly, our approach is conservative and rather underestimates the number of migrating chemicals because (1) the concentration of the analyzed migrates was rather low (chemicals migrating from 0.48 mg plastic), (2) the extraction *via* SPE probably does not recover 100% of the compounds, and (3) we only used positive ionization in the mass spectrometry.

**Table 2:** Chemical Features Detected in the Plastic Extracts and Migrates, and Tentatively Identified Compounds

	number of features						tentatively identified chemicals				
	total	extract only	low migration	readily leachable	migrate only	sum in migrate (%)^b^^c^	massbank	CPPdb	ECHAdb	NORMAN	combined (%)^c^
HDPE 1	1401	520	665	136	80	216 (15.4)	12	70	99	186	189 (13.5)
LDPE 1^a^	670	162	265	138	105	243 (36.39)	18	47	71	110	114 (17.0)
LDPE 2^a^	5923	2011	3370	515	27	542 (9.2)	41	276	402	695	715 (12.1)
LDPE 3^a^	7731	1679	2749	3244	59	3303 (42.7)	28	202	278	503	508 (6.6)
LDPE 4	6890	689	1455	4226	520	4746 (68.9)	55	374	463	912	930 (13.5)
PS 1^a^	1509	153	252	493	611	1104 (73.2)	5	45	49	148	152 (10.1)
PS 2^a^	4740	900	1203	2285	352	2637 (55.6)	81	150	211	467	485 (10.2)
PS 3^a^	278	75	150	44	9	53 (19.1)	0	4	8	16	16 (5.8)
PS 4	523	206	300	14	3	17 (3.3)	5	17	26	48	48 (9.2)
PP 1^a^	7036	1056	2586	2882	512	3394 (48.2)	58	238	321	701	716 (10.2)
PP 2^a^	13 844	3048	6307	4040	449	4489 (32.4)	99	404	549	1010	1025 (7.4)
PET 1^a^	1664	771	838	48	7	55 (3.3)	38	112	150	260	269 (16.2)
PVC 1^a^	11 791	2261	5086	4396	48	4444 (37.7)	89	441	588	1198	1213 (10.3)
PVC 2	4983	866	1854	2169	94	2263 (45.4)	51	182	277	540	557 (11.2)
PVC 3	4952	1514	2574	750	114	864 (17.4)	47	192	251	481	495 (10.0)
PVC 4	11 252	2530	5274	3198	250	3448 (30.6)	110	423	556	1171	1206 (10.7)
PUR 1	10 809	1159	2434	6795	421	7216 (66.8)	29	203	259	559	573 (5.3)
PUR 2	8627	493	535	7280	319	7599 (88.1)	11	69	90	195	199 (2.3)
PUR 3	15 815	3032	4102	8522	159	8681 (54.9)	80	279	375	942	965 (6.1)
PUR 4	3376	1014	1423	642	297	939 (27.8)	24	131	188	347	351 (10.4)
PLA 1^a^	4891	2429	2409	53	0	53 (1.1)	15	78	92	315	329 (6.7)
PLA 2^a^	5147	2654	2415	59	19	78 (1.5)	18	76	102	276	290 (5.6)
PLA 3	12 122	1894	2424	7212	592	7804 (64.4)	53	284	365	795	826 (6.8)
PLA 4^a^	6039	2959	2905	139	36	175 (2.9)	23	108	151	366	379 (6.3)

a Food contact materials.

b Sum of readily leachable and migrate only.

c % of total.

**Figure 3:**
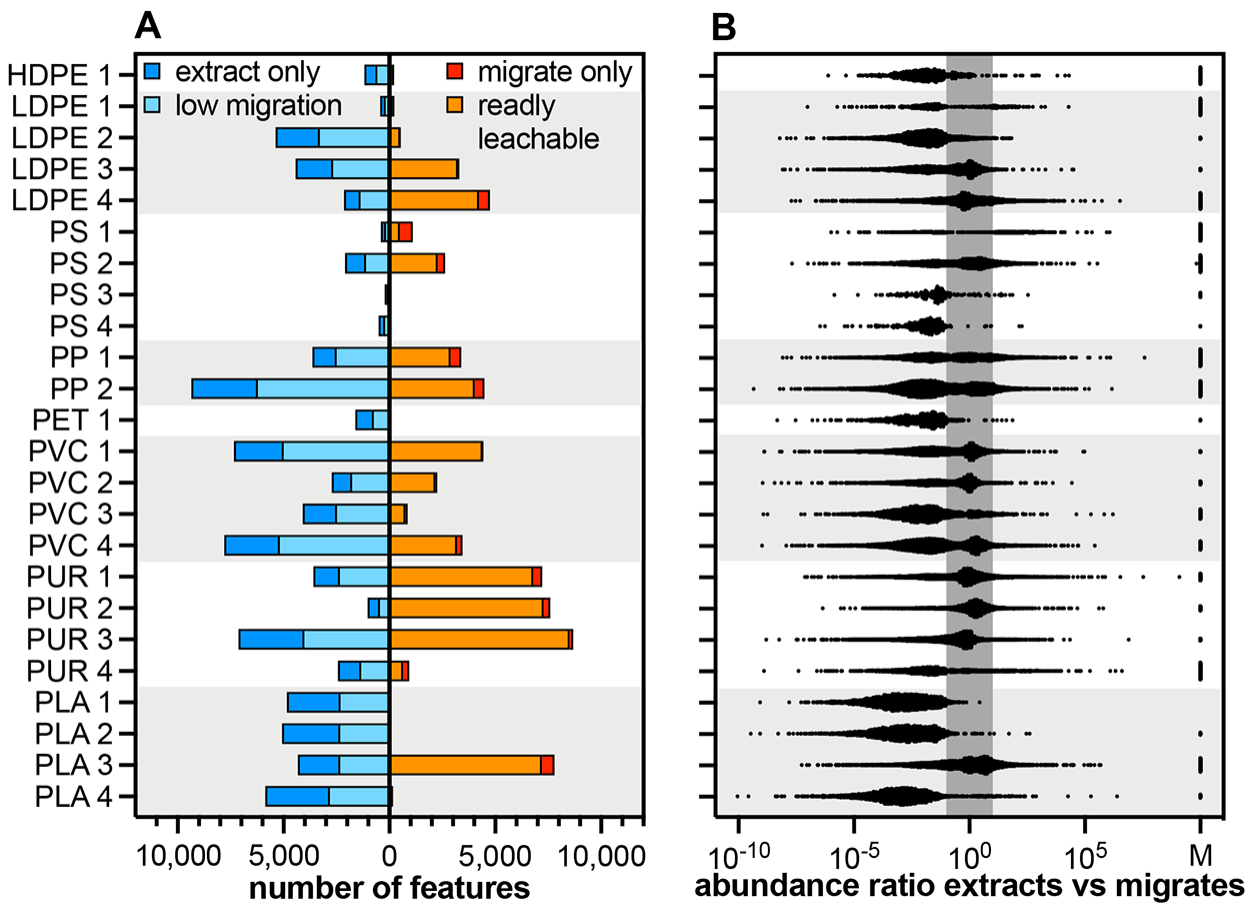
Numbers of chemical features migrating from plastic products (A) and ratios of the abundance of each feature in the extract and the migrate (B). The left side of each graph represents features with no/low migration (migration ratio of <0.1), the right side represents features that are readily leachable, that is, they are only detected in migrates (M in *B*) or migrating with a ratio of >0.1. The dark gray band in (B) highlights the area in which abundance of features is similar (maximally 10-fold lower or higher) in the extracts and migrates.

Some of the plastic products leached very few chemicals (PLA 1 < PLA 2 < PLA 4 < PS 4 = PET 1 < LDPE 2, Table 2, Figure 3A). In these samples, less than 10% of all detected features were readily leachable or newly formed in the migrates. On the other end of the spectrum, more than half of all features detected in a sample leached from PUR 3, PS 2, PLA 3, PUR 1, LDPE 4, PS 1, and PUR 2. In the latter sample, 88.1% of all features (7599 out of 8627) were present in water after 10 days of migration. As in our previous work,^4^ there was no clear association of the number of migrating compounds with the polymer type: Products made of PE, PS, PET, and PLA leached relatively few chemicals while those made of PP, PVC, and PUR leached many. However, there were notable exceptions, including LDPE 4, PS 2, and PLA 3 (many features in the migrates), as well as PVC 3 and PUR 4 (few features), making it impossible to generalize.

Taking the abundance of a feature as a proxy of its quantity, many of the migrating compounds are detected in similar levels in the extracts and the migrates, indicating they are readily leachable in water (Figures 3B and S10). The abundance of many migrating features falls in a band of 10-fold higher to 10-fold lower than in the extracts (*e.g*., in LDPE 3, LDPE 4, PVC 1, PVC 2, PUR 1, PUR 2, PUR 3, PLA 3). However, there were also several features that we detected in much higher levels in the migrates than in the extracts, no matter the polymer type (*e.g*., in LDPE 4, PP 2, PUR 1, PLA 3). This implies a preferential migration into water over methanol or an additional formation during migration. Nonetheless, these results have to be interpreted with caution given that the abundance of a feature in the mass spectroscopy may not be linearly related to its concentration.^42^

### 3.6 Most Plastic Chemicals Remain Unknown

By cross-referencing with the MassBank library and *in silico* fragmenting the compounds in the databases of Chemicals associated with Plastic Packaging (CPPdb), chemicals registered under REACH (ECHAdb), and the NORMAN Suspect List Exchange (NORMANdb), we tentatively identified 2979 unique compounds present in and/or migrating from the plastic products. This represents approximately 8% of all detected features. Only 211 compounds were identified using the empirical spectra in the MassBank library. Most of the chemicals were identified using the NORMANdb (4122 compounds). The CPPdb had the best coverage with 14.1% of the compounds in that database being detected in the analyzed plastic products. Interestingly, only 452 chemicals were covered by the ECHAdb, that is, they are registered under the European REACH regulation.

In each individual sample, we identified between 16 (PS 3) and 1213 (PVC 1) chemicals (Table 2). Generally, the identification rates (number of tentatively identified compounds out of all features detected in a sample) we achieved were low, ranging from 2.3% in PUR 2 to 17.0% in LDPE 1. This demonstrates that most plastic chemicals remain unknown. Similarly, another study analyzed the compounds migrating from plastic and glass jars and found that 99% remained unidentified.^43^ The low identification rates are even more true given that our approach may result in many false-positive annotations as indicated by the identification of a number of implausible compounds in plastics (*e.g*., pharmaceuticals). The reasons for the low performance of compound annotation are manifold: first and foremost, NIAS are prevalent, if not predominant, in plastics but not well covered in spectral libraries because of the lack of scientific attention and the unavailability of authentic standards. This is supported by the fact that only a few compounds were identified when using the MassBank library. Second, poorly fragmented chemicals might result in database hits based on few, generic fragments. Third, the MetaScope algorithm used for *in silico* fragmentation might produce false-positive hits. While we used a very extensive database search to identify leaching plastic chemicals, the compound annotations we achieved need to be interpreted in light of these limitations.

The ten most frequently identified chemicals across all plastic products include the adhesive mono(2-acryloyloxyethyl) succinate, the processing aid pentaethylene glycol, and the solvent solketal amongst other compounds known to be used in plastics (Table S9). Interestingly, all these compounds were identified multiple times in the same sample, either because of the presence of isomers or of false-positive annotations. The same is true for compounds detected across multiple polymers. While the presence of the same chemicals in multiple polymers may be counterintuitive, these compounds might represent common impurities introduced during the manufacturing process (*e.g*., in lubricants used during molding).

We prioritized the 10 features with the highest abundance in each plastic migrate. Out of a total of 240 features, we tentatively identified 45 chemicals, including multiple carboxylic acids, alcohols, and amides (Table S10). Interestingly, the organophosphates migrating from PVC products were the only tentatively identified chemicals that are obviously related to plastic additives. In these cases, the detected compounds are probably degradation products of the flame-retardant tris(2-butoxyethyl) phosphate (TBEP, migrating from PVC 4). In addition to these plastic additives, another two compounds are associated with plastic packaging according to Groh et al.,^2^ including 2-[2,2-bis(2-prop-2-enoyloxyethoxymethyl)butoxy]ethyl prop-2-enoate (ethoxylated trimethylolpropane triacrylate) and N-[3,5-bis(2,2-dimethylpropanoylamino)phenyl]-2,2-dimethylpropanamide (tris(2,2-dimethylpropionylamino)benzene).

Although only tentative (level 3 according to Schymanski et al.,^44^) the identification of these compounds appears plausible, especially for those covered by the CPPdb. Importantly, and in a more general context, we assume that very little, if any, (eco)toxicological information is available regarding the chemicals we tentatively identified here. Accordingly, the hazard of many plastic chemicals humans and wildlife are likely exposed to remains unknown and, thus, unregulated.

Our study highlights that plastic products leach chemicals triggering toxicity. While the prevalent baseline toxicity points toward unspecific effects relevant in an environmental rather than a human health context, the prevalent antiandrogenicity is an indicator for the leaching of endocrine-disrupting chemicals relevant for human health. Our results also show that many more chemicals are migrating from plastics than previously known. The large number of compounds, and the fact that most of these remain unidentified, pinpoint the shortcomings of current scientific and regulatory approaches to the chemicals leaching from plastics. As an example, very few of the chemicals we found migrating from plastic products marketed in the European Union are covered by REACH. Accordingly, these compounds do not undergo formal risk assessment and it, thus, remains unknown whether many chemicals leaching from consumer plastics are safe. To address these regulatory gaps, the combination of whole migrate toxicity testing and nontarget chemical analysis used in this study represent a way forward since it allows benchmarking the toxicity of chemicals migrating from the final product. In addition, this approach enables the identification and prioritization of new, potentially toxic compounds to further quantify actual exposures and health hazards *in vivo*. While further research is always warranted, the regulatory community needs to prioritize the issue of plastic chemicals and develop conceptual approaches to address the high number of leaching compounds. At the same time, manufacturers can improve the chemical safety of plastics. For instance, the chemical composition of plastics can be simplified by reducing the number of starting substances and additives and by better controlling polymerization and processing. Another approach would be to keep plastics chemically complex but significantly reduce the migration by covalently binding additives to the polymer backbone, reducing the diffusion coefficient of the polymer, or introducing additional barrier functions. In any case, such improvements require a fundamental rethinking and redesign of the plastics we are using today.

## Supporting Information Available

Methodology and results to determine migration conditions, methodology chemical analysis, information on databases used for compound identification, further *in vitro* toxicity data of migrates and aqueous migrates (baseline toxicity, oxidative stress, estrogenicity, antiandrogenicity) of reference compounds and samples; toxicity of migrates *vs* nonextracts; toxicity of migrates (with SPE) *vs* aqueous migrates (without SPE); tentatively identified compounds (most frequent across all samples, with highest abundances); and abundance of chemical features detected in migrates *vs* extracts (PDF)
