# Supplementary material for: Plastic Products Leach Chemicals That Induce In Vitro Toxicity under Realistic Use Conditions
Source: Environ Sci Technol. 2021 Aug 17;55(17):11814–23. doi: 10.1021/acs.est.1c01103 (PMC8427741; doi:10.1021/acs.est.1c01103)
Supplement: Supplementary file 1 — es1c01103_si_001.pdf [file es1c01103_si_001.pdf]

Supporting Information

Plastic products leach chemicals that induce *in vitro* toxicity under realistic use conditions

Lisa Zimmermann[†], Zdenka Bartosova[‡], Katharina Braun[†], Jörg Oehlmann[†], Carolin Völker[+, §]

Martin Wagner[*, +, ‡]

[†]Goethe University Frankfurt am Main, Department Aquatic Ecotoxicology, Max-von-Laue-Str. 13, 60438, Frankfurt, Germany

[‡]Norwegian University of Science and Technology (NTNU), Department of Biology, Høgskoleringen 5, 7491 Trondheim, Norway

[§]Institute for Social-Ecological Research, Hamburger Allee 45, 60486 Frankfurt am Main, Germany

[+]Both authors contributed jointly

[*]Corresponding author. M. Wagner. Norwegian University of Science and Technology (NTNU), Department of Biology, Høgskoleringen 5, 7491 Trondheim, Norway. Email: martin.wagner@ntnu.no

DOI of the main article: 10.1021/acs.est.1c01103

Total number of pages: 28

Total number of tables: 10

Total number of figures: 10

Supplementary methods

S1. Determination of migration conditions

In order to determine migration conditions, 60 g of PVC 4 were placed in 2 L-glass bottle containing 1.5 L ultrapure water and incubated for two, five or ten days (d) in a climate chamber (Thermotec GmbH & Co. KG, Weilburg) at 40 °C in the dark. Three mL of the aqueous solution were stored in clean brown glass vials with PFTE caps at 8 °C until application to the bioassay. The remaining volume was transferred into new 2-L glass bottles for solid-phase extraction (SPE). Of procedural and SPE blanks, only some induced a very low baseline toxicity. Neither effects of aqueous migrates nor migrates (SPE-extracted) on unspecific and endocrine endpoints correlated with the leaching time (Table S1). Instead, effect concentrations (ECs) and levels (ELs) rather depended on the endpoint and whether the sample was extracted by SPE or not. Exemplary, after 10 d of migration the aqueous migrate induced a stronger baseline toxicity than after 5 d whereas it was vice versa for the SPE-extracted migrate. Thus, we chose the test setup recommended by the European Commission regulation on plastic FCMs of 10 d.[1]

S2. Databases for *in silico* fragmentation

We generated three databases for the tentative identification of plastic chemicals using the Metascope algorithm in Progenesis QI:

1) the mapped version of the database of Chemicals associated with Plastic Packaging (CPPdb) containing chemicals likely[2] and possibly[3] associated with plastic packaging,

2) the chemicals registered under the REACH regulation in 2020 (ECHAdb) falling in all product categories except PC 11 (Explosives), PC 12 (Fertilizers), PC 14 (Metal surface treatment products), PC 23 (Leather treatment products), PC 25 (Metal working fluids), PC 27 (Plant protection products), PC 29 (Pharmaceuticals), PC 33 (Semiconductors), PC 36 (Water softeners), PC 37 (Water treatment chemicals), PC 38 (Welding and soldering products, flux products), PC 42

(Electrolytes for batteries). We downloaded the lists from https://echa.europa.eu/information-on-chemicals/registered-substances on 13.12.2020, combined all available CAS numbers and removed duplicates,

3) the chemicals (pre)registered under the REACH regulation in 2017 (NORMANdb) as provided by the NORMAN Suspect List Exchange.[4] We combined all available SMILES codes and removed duplicates.

To generate molecular structures for *in silico* fragmentation, we annotated the available CAS numbers of the chemicals in the ECHAdb with SMILES codes using the CompTox Chemicals Dashboard (https://comptox.epa.gov/dashboard/dsstoxdb/batch_search). For the CPPdb and the NORMANdb, we used the SMILES codes available in the respective list. We converted the SMILES to CID numbers using PubChem's Identifier Exchange Service (https://pubchem.ncbi.nlm.nih.gov/idexchange) and downloaded the structural information as individual sdf file per database using PubChem Entrez (https://pubchem.ncbi.nlm.nih.gov). Each annotation step resulted in the loss of compounds that either had no SMILES codes, CID or structural information (Table S2). The resulting structural databases contained 2680 (CPPdb), 7092 (ECHAdb), and 65,738 chemicals (NORMANdb) for *in silico* fragmentation.

Tables and Figures

Table S1. Determination of migration conditions using PVC 4.

Sample	Test	Endpoint	Migrate (SPE-extracted)			Aqueous Migrate	
			Day 2	Day 5	Day 10	Day 5	Day 10
PVC 4	Microtox	EC_{20} [mg plastic well^{-1}]	4.17	2.05	4.17	0.71	0.43
		EC_{50} [mg plastic well^{-1}]	17.10	9.46	14.01	—	1.66
		LI [%]	100 .0	100.0	100.0	45.70	59.55
	AREc32	EC_{IR2} [mg plastic well^{-1}]	10.33	10.90	11.93	n.a.	n.a
		IR [%]	77.26	77.76	60.56	n.a.	n.a
		Cytotoxic concentration [mg plastic well^{-1}]	n.a.	n.a	200.0	200.0	20 0
	YES	Relative estrogenic activity [%]	—	—	—	n.a.	96.35
		Cytotoxicity, EC_{20} [mg plastic well^{-1}]	yes	-	31.84	52.44	98.94
	YAAS	Relative antiandrogenic activity [%]	60.68	50.11	50.10	—	—
		EC_{50} [mg plastic well^{-1}]	39.51	46.15	50.34	n.a.	n.a.
		Cytotoxicity, EC_{20} [mg plastic well^{-1}]	—	—	70.68	88.11	80.45
PB	Microtox	EC_{20} [mg plastic well^{-1}]	503.8	145.9	289.8	—	—
		EC_{50} [mg plastic well^{-1}]	—	—	501.8		
		LI [%]	21.82	42.77	53.35	—	—
	AREc32	EC_{IR2} [mg plastic well^{-1}]	—	—	—	n.a.	n.a
		IR [%]	1.55	1.57	1.62	n.a.	n.a
		Cytotoxic concentration [mg plastic well^{-1}]	n.a.	n.a	—	—	—
	YES	Relative estrogenic activity [%]	—	—	—	n.a.	n.a.
		Cytotoxicity, EC_{20} [mg plastic well^{-1}]	yes	yes	—	—	—
	YAAS	Relative antiandrogenic activity [%]	—	—	—	—	—
		Cytotoxicity, EC_{20} [mg plastic well^{-1}]	—	—	—	—	—
SPE blank	Microtox	EC_{20} [mg plastic well^{-1}]	—	423.3	—	—	—
		EC_{50} [mg plastic well^{-1}]	—	—	—	—	—
		LI [%]	16.63	31.32	18.83	0.50	—
	AREc32	EC_{IR2} [mg plastic well^{-1}]	1.32	1.46	1.37	n.a.	n.a
		IR [%]	—	—	—	n.a.	n.a
		Cytotoxic concentration [mg plastic well^{-1}]	n.a.	n.a	—	—	—
	YES	Relative estrogenic activity [%]	—	—	—	n.a.	n.a.
		Cytotoxicity, EC_{20} [mg plastic well^{-1}]	yes	yes	—	—	—
	YAAS	Relative antiandrogenic activity [%]	—	—	—	—	—
		Cytotoxicity, EC_{20} [mg plastic well^{-1}]	—	—	—	—	—

Note: PVC, polyvinyl chloride; PB, procedural blank, SPE, solid-phase-extraction; IR, induction ratio; $EC_{20/50}$, effective concentration inducing 20/50% effect; LI, luminescence inhibition, EC_{IR2}, effective concentration leading to a luciferase induction ratio of 2.0 over the control (IR 2); —, no effect at measured concentrations; n.a., not assessed (e.g., due to cytotoxicity).

Number of experiments (*n*) performed and concentrations analyzed in 1:2 serial dilutions/assay:

Microtox: aqueous migrate n = 1 in duplicates, 0.018–5.00 mg plastic well^{-1}, migrate: n = 2–4 in duplicates, 0.018–600 mg plastic well^{-1}.

AREc32 assay: migrate n = 3–4 in duplicates, 1.56–200 mg plastic well^{-1}, IRs are given for the highest measured noncytotoxic concentration.

YES: aqueous migrate: n = 8, 3 mg plastic well^{-1}, migrate: n = 8–16, 0.195–100 mg plastic well^{-1}, relative activities are given for the highest measured noncytotoxic concentration.

YAAS: aqueous migrate: n = 8, 3.00 mg plastic well^{-1}, migrate: n = 16–24, 0.195–100 mg plastic well^{-1}, limit of detection = 57.6, relative activities are given for the highest measured noncytotoxic concentration.

Table S2. Number of compounds covered by the three databases used for the tentative identification of plastic chemicals using *in silico* fragmentation.

Database	CAS numbers	SMILES	CIDs	Structures for *in silico* fragmentation
CPPdb	4256[a]	2777	2702	2680
ECHAdb	10,443	7305	7099	7092
NORMANdb	68,679[a]	68,679	67,628	65,738

[a] we used the SMILES codes directly

Table S3. Reference compounds used in the *in vitro* bioassays: 3,5-dichlorophenol for luminescence inhibition of *A. fischeri*, 17*β*-estradiol for agonistic activity at the hER*α*, and flutamide for antagonistic activity at the hAR as well as *tert*-butylhydroquinone (t-BHQ) for induction of an oxidative stress response. For 3,5-DCP, mean EC_{50} values and r^2 are derived from 13 (migrate) and 7 (aqueous migrate), for 17*β*-estradiol and flutamide from 19 and 22 independent experiments, and for t-BHQ from one test.

Reference compound	Grade, supplier, CAS number	Concentration range (mol L^{-1})	EC_{50} (mol L^{-1})	r^2
3,5-DCP (migrate)	97 %, Sigma-Aldrich, 591-35-5	1.7×10^{-6} – 2.9×10^{-4}	3.65×10^{-5}	0.993
3,5-DCP (aqueous migrate)	97 %, Sigma-Aldrich, 591-35-5	4.6×10^{-6} – 5.9×10^{-4}	8.01×10^{-5}	0.995
17β-estradiol	> 99 %, Merck, 50-28-2	1.0×10^{-12} – 1.0×10^{-8}	7.63×10^{-11}	0.989
Flutamide	> 98 %, Merck, 13311-84-7	7.8×10^{-7} – 5.0×10^{-5}	1.24×10^{-5}	0.969
t-BHQ	97 %, Sigma-Aldrich, 1948-33-0	2.0×10^{-6} – 5.6×10^{-5}	—	0.928

Table S4. Baseline toxicity (mean ± SEM) of procedural (PB) and SPE blanks (SPE) as well as of plastic migrates in the Microtox assay.

Sample	EC_{20} (mg plastic well^{-1})	EC_{50} (mg plastic well^{-1})	Luminescence inhibition (%)[a]	n[b]
PB1	—	—	16.1 ± 2.97	6
PB2	—	—	19.5 ± 1.45	6
PB3	—	—	17.8 ± 1.54	3
PB4	—	—	18.5 ± 1.72	3
PB 5	—	—	4.30 ± 1.61	3
PB 6	—	—	3.70 ± 1.78	3
SPE 1	—	—	20.1 ± 1.61	6
SPE 2	—	—	11.9 ± 1.59	3
SPE 3	—	—	3.23 ± 2.25	3
HDPE 1	53.2 ± 13.9	271 ± 11.4	66.4 ± 1.52	3
LDPE 1	2.55 ± 0.49	8.44 ± 1.37	99.5 ± 0.34	4
LDPE 2	2.83 ± 1.44	12.8 ± 2.37	100 ± 0.00	3
LDPE 3	22.6 ± 5.06	127 ± 14.78	79.5 ± 1.48	3
LDPE 4	3.38 ± 0.39	11.0 ± 1.23	100 ± 0.00	3
PS 1	17.7 ± 1.86	56.4 ± 3.72	99.4 ± 0.34	3
PS 2	0.48 ± 0.28	3.37 ± 0.79	100 ± 0.00	3
PS 3	322 ± 77.1	—	31.7 ± 1.48	3
PS 4	120 ± 17.1	—	47.2 ± 0.14	3
PP 1	22.2 ± 3.82	53.1 ± 3.78	100 ± 0.00	3
PP 2	4.24 ± 0.22	31.7 ± 2.29	100 ± 0.00	3
PET 1	10.6 ± 1.55	37.8 ± 7.23	91.7 ± 1.08	2
PVC 1	0.56 ± 0.14	1.98 ± 0.53	100 ± 0.00	6
PVC 2	0.14 ± 0.06	1.59 ± 0.63	100 ± 0.00	6
PVC 3	0.93 ± 0.72	5.06 ± 2.46	99.3 ± 0.49	5
PVC 4	0.12 ± 0.03	2.72 ± 0.99	99.5 ± 0.30	6
PUR 1	23.2 ± 0.62	93.8 ± 3.17	89.4 ± 0.74	3
PUR 2	2.04 ± 0.64	11.3 ± 1.67	98.4 ± 0.69	4
PUR 3	32.7 ± 5.29	122 ± 13.4	86.9 ± 1.69	4
PUR 4	2.63 ± 1.10	15.5 ± 3.48	100 ± 0.04	4
PLA 1	109 ± 7.08	247 ± 10.1	81.1 ± 0.26	3
PLA 2	47.4 ± 4.37	270 ± 17.8	63.7 ± 1.42	3
PLA 3	3.98 ± 0.62	16.7 ± 1.20	100 ± 0.00	3
PLA 4	143 ± 7.66	—	46.7 ± 1.96	3

Note: —, no luminescence inhibition observed (< 20% for EC_{20} or ≤ negative controls for luminescence inhibition) at analyzed concentrations.

[a] Luminescence inhibition induced by migrates from 600 mg plastic well^{-1}.

[b] Number of independent experiments performed with two technical replicates, each.

Table S5. Oxidative stress response (mean ± SEM) as well as cytotoxicity induced by procedural (PB) and SPE blanks (SPE) as well as plastic migrates in the AREc32 assay.

Sample	EC_{IR2} (mg plastic well^{-1})	Max. IR[a]	Noncytotoxic conc. (mg plastic well^{-1})[b]	n[c]
PB1	—	0.91 ± 0.11	200	3
PB2	—	0.87 ± 0.09	200	3
PB3	—	1.00 ± 0.04	200	3
PB4	—	1.25 ± 0.11	200	3
PB 5	—	1.02 ± 0.21	200	3
PB 6	—	0.83 ± 0.09	200	3
SPE 1	—	0.84 ± 0.07	200	3
SPE 2	—	1.24 ± 0.01	200	3
SPE 3	—	1.05 ± 0.15	200	3
HDPE 1	—	1.03 ± 0.07	6.25	3
LDPE 1	12.0 ± 1.06	58.9 ± 16.0	50	3
LDPE 2	60.6 ± 1.46	2.45 ± 0.17	100	3
LDPE 3	64.8 ± 8.38	9.81 ± 1.01	200	3
LDPE 4	2.15 ± 0.25	46.70± 15.9	6.26	3
PS 1	9.64 ± 0.38	80.3 ± 9.58	50	3
PS 2	87.3 ± 141	1.91 ± 0.17[d]	6.25	3
PS 3	97.2 ± 57.3	3.49 ± 0.37[e]	200	3
PS 4	107 ± 14.6	3.30 ± 0.29	200	3
PP 1	3.20 ± 0.18	43.9 ± 3.55	12.5	3
PP 2	7.55 ± 0.34	6.18 ± 0.46	50	3
PET 1	131 ± 29.1	5.67 ± 1.68	200	3
PVC 1	6.62 ± 2.65	24.4 ± 17.6	12.5	3
PVC 2	91.6 ± 79.2	10.1 ± 8.39	25	3
PVC 3	74.0 ± 10.0	5.84 ± 1.03	200	3
PVC 4	18.8 ± 12.0	29.4 ± 13.8	50	3
PUR 1	25.0 ± 0.48	10.2 ± 0.97	100	3
PUR 2	12.9 ± 3.41	4.57 ± 0.56	50	3
PUR 3	15.2 ± 2.96	23.8 ± 1.22	100	3
PUR 4	19.5 ± 3.70	9.87 ±0.41	100	3
PLA 1	103.5 ± 11.5	4.19 ± 0.49	200	3
PLA 2	110.7 ± 9.08	4.56 ± 1.03	200	3
PLA 3	26.9 ± 2.41	2.89 ± 0.90	50	3
PLA 4	—	1.36 ± 0.21	100	3

Note: —, no oxidative stress response observed (induction rate < 2) at the analyzed concentrations.

[a] Maximal luciferase induction ratio induced by the highest measured non-cytotoxic concentration.

[b] Highest analyzed concentration at which no cytotoxicity was observed. Concentrations between 1.56 and 200 mg plastic well^{-1} were tested.

[c] Number of independent experiments performed in two technical replicates, each.

[d] IR of two replicates exceeded 2 and thus, the EC_{IR2} was still calculated using all 3n.

[e] Only of n = 2 since 1n was cytotoxic at 200 mg plastic well^{-1} and thus, excluded.

Table S6. Estrogenic and antiandrogenic activities (mean ± SEM) of procedural blanks (PB), SPE blanks (SPE) and samples in YES and YAAS as well cytotoxicity.

	YES				YAAS				
Sample	Cytotox.[a]	rEA (%)[b]	*p* value[c]	*n*	Cytotox.[a]	rAA (%)[b]	EC_{50} (mg plastic)[d]	*p* value[c]	*n*
PB1	—	0.08 ± 0.05	n.a.	24	—	—	—	n.a.	32
PB2	—	0.15 ± 0.13	n.a.	24	—	10.4 ± 1.99	—	n.a.	32
PB3	—	0.44 ± 0.07	n.a.	24	—	4.76 ± 1.41	—	n.a.	32
PB4	—	0.18 ± 0.13	n.a.	24	—	14.5 ± 2.18	—	n.a.	24
PB 5	—	0.14 ± 0.05	n.a.	24	—	1.97 ± 1.29	—	n.a.	32
PB 6	—	0.10 ± 0.04	n.a.	24	—	7.58 ± 0.98	—	n.a.	32
SPE 1	—	—	n.a.	24	—	—	—	n.a.	32
SPE 2	—	0.24 ± 0.21	n.a	24	—	—	—	n.a.	32
SPE 3	—	0.14 ± 0.18	n.a.	24	—	—	—	n.a.	32
HDPE 1	—	0.51 ± 0.97	n.a.	16-24		92.1 ± 0.50	23.4	<0.001	32
LDPE 1	49.0	— (25.0)	n.a.	16-24	83.9	32.6 ± 1.62 (50.0)	—	<0.001	24
LDPE 2	57.0	1.04 ± 0.08 (50.0)	n.a.	16-24	51.8	— (25.0)	—	n.a.	24
LDPE 3	—	0.75 ± 0.08	n.a.	16-24	—	71.6 ± 1.49	63.5	<0.001	24
LDPE 4	100	0.50 ± 0.01 (50.0)	n.a.	16-24	—	91.6 ± 0.56	36.7	<0.001	24
PS 1	—	—	n.a.	16-24	—	61.6 ± 2.00	72.3	<0.001	32
PS 2	3.33	0.82 ± 0.20 (3.13)	n.a.	16-24	0.35	—[f]	—	n.a.	32
PS 3	—	0.14 ± 0.06	n.a.	16-24	—	0.72 ± 1.21	—	n.a.	32
PS 4	—	0.84 ± 0.11	n.a.	16-24	—	7.07 ± 1.73	—	n.a.	32
PP 1	75.7	— (50.0)	n.a.	16-24	19.9	40.6 ± 1.03 (25.0)	—	<0.001	32
PP 2	43.0	— (25.0)	n.a.	16-24	0.03	— (0.01)	—	n.a.	24
PET 1	—	—	n.a.	16-24	—	1.13 ± 1.22	—	n.a.	24
PVC 1	21.3	0.46 ± 0.12 (12.5)	n.a.	16-24	60.0	54.0 ± 2.53 (50.0)	47.0	<0.001	32
PVC 2	2.02	59.4 ± 2.38 (1.56)[e]	<0.001	16-24	1.66	90.9 ± 1.25 (0.78)	0.28	<0.001	32
PVC 3	75.6	0.21 ± 0.16 (50)	n.a.	16-24	3.66	— (0.31)	—	n.a.	32
PVC 4	41.0	0.98 ± (25)	n.a.	16-24	40.1	19.3 ± 3.00 (25.0)	—	n.a	32
PUR 1	—	0.01 ± 0.06	n.a.	16-24	—	45.9 ± 1.68	—	<0.001.	32
PUR 2	—	0.36 ± 0.03	n.a.	16-24	—	65.8 ± 3.20	70.3	<0.001	32
PUR 3	—	0.90 ± 0.33	n.a.	16-24	—	79.9 ± 2.27	60.1	<0.001	32
PUR 4	—	0.43 ± 0.04	n.a.	16-24	—	92.2 ± 0.68	20.2	<0.001	32
PLA 1	22.1	— (12.5)	n.a.	16-24	—	42.5 ± 1.45	—	<0.001.	32
PLA 2	67.4	— (25)	n.a.	16-24	—	7.85 ± 1.95	—	n.a.	32
PLA 3	0.10	— (0.08)	n.a.	16-24	0.02	4.51 ± (0.01)	—	n.a.	32
PLA 4	—	—	n.a.	16-24	—	12.3 ± 1.46	—	n.a.	32

Note: rEA, relative estrogenic activity; rAA, relative antiandrogenic activity; —, No effect observed for the respective endpoint; n a., not analyzed.

[a] Cytotoxicity (cytotox.) as EC_{20} (mg plastic well^{-1}) of migrates from ≤ 100 mg plastic well^{-1}.

[b] rEA or rAA for the highest measured non-cytotoxic concentration (in brackets if not 100 mg well^{-1}).

[c] Statistical differences of relative activities were only analyzed if > limit of detection (YES: 1.65%, YAAS: 27.3%). *p* value compared to the control using Kruskal-Wallis with Dunn's post hoc test.

[d] For the antiandrogen activity EC_{50} values were derived if within the measured concentrations.

[e] For the estrogenic activity of PVC 2 a EC_{50} of 0.27 mg plastic well^{-1} was derived.

[f] All measured concentrations (0.20–100 mg plastic well^{-1}) were cytotoxic, such that antiandrogenicity could not be assessed

Table S7. Comparison of the toxicity of migrates and extracts.

Sample	EC_{20} baseline toxicity (mg plastic well^{-1})		EC_{IR2} oxidative stress (mg plastic well^{-1})		% relative estrogenic activity		% relative antiandrogenic activity	
	Extract	Migrate	Extract	Migrate	Extract	Migrate	Extract	Migrate
HDPE 1	14.6	—	—	—	2.75	(0.34)	31.53	(13.7)
LDPE 1	4.34	2.55	—	—	(0.28)	—	(2.16)	(0.14)
LDPE 2	1.02	2.83	—	—	(2.21)	(0.31)	(9.30)	—
LDPE 3	—	—	—	—	(1.63)	(0.01)	(11.1)	(0.35)
LDPE 4	2.63	3.38	0.48	2.15	(0.71)	—	(12.3)	(4.84)
PS 1	—	17.7	—	—	3.82	—	—	—
PS 2	1.30	0.48	2.79	—	(1.13)	—	—	—
PS 3	22.3	—	x	—	(0.37)	(0.16)	—	—
PS 4	18.3	—	x	—	(1.17)	—	(1.16)	—
PP 1	—	22.2	3.83	3.20	—	—	47.84	(0.31)
PP 2	3.63	4.24	0.99	—	—	—	—	—
PET 1	—	10.6	6.69	—	(1.22)	(0.05)	(16.3)	—
PVC 1	0.23	0.56	6.64	6.62	—	—	(25.5)	(6.05)
PVC 2	1.22	0.14	2.14	—	27.1	60.28	40.1	90.9
PVC 3	1.80	0.93	2.42	—	—	—	34.6	—
PVC 4	0.49	0.12	1.16	—	6.91	—	48.4	(2.47)
PUR 1	3.02	—	1.13	—	(0.59)	—	55.8	(0.08)
PUR 2	2.73	2.04	0.51	—	—	(0.02)	69.0	(4.37)
PUR 3	3.56	—	0.47	—	(0.36)	(0.14)	82.3	(1.65)
PUR 4	10.2	2.63	1.82	—	(2.26)	(0.07)	30.4	(10.7)
PLA 1	2.12	—	—	—	(0.02)	—	—	(1.36)
PLA 2	6.21	—	—	—	(0.34)	—	(13.2)	—
PLA 3	0.01	3.98	0.98	—	—	—	—	—
PLA 4	1.32	—	—	—	—	(0.14)	(15.3)	(8.02)

Note: –, no effect at concentrations analyzed or value not calculable.

Bioassay results of migrates and extracts at concentration ranges or points assessed for both leaching conditions: Baseline toxicity: 0—22.5 mg plastic well^{-1}, oxidative stress response induction: 0—7.5 mg plastic well^{-1}, estrogenic and antiandrogenic activities: 3.75 mg plastic well^{-1} (exception: PVC 2: 0.94 (YES) and 0.78 mg plastic well^{-1} (YAAS)). Relative activities < LOD are given in brackets and correspond to 1.65 % (migrates) and 2.33 % (extracts) for estrogenic as well as to 27.3 % (migrates) and 29.2 % (extracts) for antiandrogenic activities.

Table S8. Comparison of the toxicity of migrates (with SPE) with aqueous migrates (without SPE).

Sample	EC_{20} baseline toxicity (mg plastic $well^{-1}$)		LI baseline toxicity (mg plastic $well^{-1}$)		% relative antiandrogenic activity	
	Migrate	Aqueous migrate	Migrate	Aqueous migrate	Migrate	Aqueous migrate
HDPE 1	—	—	2.75	3.85	(11.4)	(7.18)
LDPE 1	2.55	2.88	36.1	41.1	<LOD	>LOD
LDPE 2	2.83	3.84	32.5	29.6	—	—
LDPE 3	—	—	7.45	—	(0.23)	—
LDPE 4	3.38	3.24	28.8	39.1	(3.74	(16.0)
PS 1	—	4.83	5.46	21.0	(0.01)	(0.15)
PS 2	0.48	1.10	56.3	61.7	—	(21.8)
PS 3	—	—	2.76	4.63	—	—
PS 4	—	—	1.84	1.48	—	(1.85)
PP 1	—	—	2.85	9.85	(0.13)	29.4
PP 2	4.24	3.51	21.9	30.2	—	cytotoxic
PET 1	—	4.37	10.1	23.6	—	—
PVC 1	0.56	0.46	68.5	86.1	(5.02)	(3.38)
PVC 2	0.14	0.42	67.6	72.6	cytotoxic	cytotoxic
PVC 3	0.93	0.80	59.9	47.4	—	cytotoxic
PVC 4	0.12	0.77	60.1	50.9	(1.55)	(17.9)
PUR 1	—	—	5.18	—	(0.05)	(0.39)
PUR 2	2.04	3.37	35.6	30.5	(3.61)	(3.33)
PUR 3	—	—	3.84	16.1	(1.23)	(1.48)
PUR 4	2.63	1.43	30.5	44.6	(8.31)	(16.9)
PLA 1	—	—	0.15	2.58	(1.05)	—
PLA 2	—	—	4.06	7.23	—	—
PLA 3	3.98	0.78	24.0	70.2	—	—
PLA 4	—	—	1.35	18.5	(7.87)	(0.38)

Note: Note: –, no effect at concentrations analyzed or value not calculable.

Bioassay results of migrates and extracts at concentration ranges or points assessed for both leaching conditions: Baseline toxicity: EC_{20} at 0—5.0 mg plastic $well^{-1}$ and luminescence inhibition (LI) at 3 mg plastic $well^{-1}$, antiandrogenic activities: 3.00 mg plastic $well^{-1}$ (exception: PVC 2: 0.78 mg plastic $well^{-1}$). Relative antiandrogenic activities < LOD are given in brackets and correspond to 27.3 % (migrates) and 29.2 % (extracts)..

Table S9. The ten most frequently identified compounds across all plastic samples. Note that the compounds have been identified multiple times in one sample, indicating the presence of isomers or false-positive annotations.

PubChem CID	No. IDs	Common name	IUPAC name	Use according to PubChem	Formula	m/z	Detected in samples
175956	77	Mono(2-acryloyloxyethyl) succinate	4-oxo-4-(2-prop-2-enoyloxyethoxy)butanoic acid	adhesive	C9H12O6	455.1163	PLA 1, PLA 2, PLA 3, PLA 4
17083	40	–	11-aminoundecanoic acid	used in plastics[a]	C11H23NO2	184.1703	HDPE 1, LDPE 1, LDPE 3, PET 1, PLA 3, PLA 4, PP 2, PS 2, PUR 1, PUR 2, PUR 3, PUR 4, PVC 2, PVC 4
62551	40	Pentaethylene glycol	2-[2-[2-[2-(2-hydroxyethoxy)ethoxy] ethoxy]ethoxy]ethanol	processing aid and additive[b]	C10H22O6	221.1381	LDPE 3, LDPE 4, PLA 2, PLA 3, PP 1, PS 2, PUR 4, PVC 1, PVC 4
9988	39	–	ethyl 2-fluoroacetate	no information	C4H7FO2	107.0500	HDPE 1, LDPE 1, LDPE 2, LDPE 3, LDPE 4, PLA 2, PP 2, PS 2, PS 4, PUR 1, PUR 2, PUR 3, PUR 4, PVC 1, PVC 3, PVC 4
69661	37	12-Aminolauric acid	12-aminododecanoic acid	used in plastics[a]	C12H25NO2	198.1857	HDPE 1, LDPE 1, LDPE 3, PET 1, PLA 3, PLA 4, PP 3, PS 4, PUR 1, PUR 2, PUR 3, PUR 4, PVC 2
242516	37	6-Deoxy-D-mannono-4-lactone	3,4-dihydroxy-5-(1-hydroxyethyl)oxolan-2-one	no information	C6H10O5	145.0499	PLA 1, PLA 2, PLA 3, PLA 4, PS 2, PUR 1
20836325	34	–	(2E,6Z)-dodeca-2,6-dien-1-ol	no information	C12H22O	165.1642	HDPE 1, LDPE 1, LDPE 2, LDPE 3, PET 1, PLA 1, PLA 2, PP 1, PS 1, PS 2, PS 4, PUR 1, PVC 1, PVC 2, PVC 3, PVC 4
7528	33	Solketal	(2,2-dimethyl-1,3-dioxolan-4-yl) methanol	solvent[b]	C6H12O3	265.1641	LDPE 3, LDPE 4, PLA 3, PP 1, PP 2, PVC 4
13690	32	Laurolactam	Azacyclotridecan-2-one	used in plastics[a]	C12H23NO	198.1858	HDPE 1, LDPE 2, LDPE 3, PET 1, PP 2, PS 2, PUR 1, PUR 4, PVC 2, PVC 3
67796	31	–	hexanoyl fluoride	no information	C6H11FO	119.0868	HDPE 1, LDPE 2, LDPE 3, PET 1, PLA 1, PLA 4, PP 2, PS 2, PUR 4, PVC 2, PVC 4

[a] according to EPA CPDat Chemical and Product Categories, [b] according to EPA Safer Choice

Table S10. Tentatively identified compounds out of the ten features with the highest abundance in the migrate of each plastic sample.

Sample	PubChem CID	Formula	Score	Description	Neutral mass (Da)	m/z	Retention time (min)
HDPE 1	none identified						
LDPE 1	72822400	C29H36O8	41.3	6-[2-(11,17-dihydroxy-10,13-dimethyl-3-oxo-7,8,9,11,12,14,15,16-octahydro-6H-cyclopenta[a]phenanthren-17-yl)-2-oxoethoxy]carbonylcyclohex-3-ene-1-carboxylic acid	512.2415	535.2307	24.97
LDPE 2	548230	C14H28O6	41.1	2-(hydroxymethyl)-6-octoxyoxane-3,4,5-triol		315.1783	20.06
LDPE 2	88669	C16H20N4O2	40.1	2-[4-[(4-aminophenyl)diazenyl]-N-(2-hydroxyethyl)anilino]ethanol		323.1475	20.06
LDPE 2	103841	C16H32O6	40.8	2-[2-[2-(2-hydroxyethoxy)ethoxy]ethoxy]ethyl 2-ethylhexanoate	320.2209	343.2101	23.54
LDPE 2	92926	C13H26O4	42.6	2,3-dihydroxypropyl decanoate	246.1840	269.1732	23.73
LDPE 2	73743781	C26H40NO5-	40.9	3-[2-[2-(2-hydroxyethyl)anilino]ethoxycarbonyl]pentadec-5-enoate	446.2911	469.2803	25.49
LDPE 2	99120788	C18H36O6	42.3	(2R,3S,4R,5R)-6-dodecoxy-2,3,4,5-tetrahydroxyhexanal	348.2523	371.2416	25.56
LDPE 3	3218	C17H26O4	45.2	2,5-dihydroxy-3-undecylcyclohexa-2,5-diene-1,4-dione	294.1836	277.1804	23.88
LDPE 4	21149416	C18H30O7	41.3	6-(5-carboxy-2,2,3-trimethylpentanoyl)oxy-4,5,5-trimethyl-6-oxohexanoic acid	358.1991	381.1883	20.14
LDPE 4[a]	115157	C21H32O9	42.3	2-[2,2-bis(2-prop-2-enoyloxyethoxymethyl)butoxy]ethyl prop-2-enoate	428.2051	451.1943	21.97
LDPE 4	139595991	C21H32O8	44	1-[3-(2-carboxycyclohexanecarbonyl)oxy-2,2-dimethylpropoxy]carbonylcyclohexane-1-carboxylic acid	412.2109	435.2001	22.42
PS 1	446541	C17H20O6	40.4	(E)-6-(4-hydroxy-6-methoxy-7-methyl-3-oxo-1H-2-benzofuran-5-yl)-4-methylhex-4-enoic acid		353.1582	18.88
PS 1[a]	115157	C21H32O9	42.9	2-[2,2-bis(2-prop-2-enoyloxyethoxymethyl)butoxy]ethyl prop-2-enoate	428.2049	451.1941	21.98
PS 2	74338	C21H20O3	42.2	[3,4-bis(phenylmethoxy)phenyl]methanol	320.1407	343.1299	9.56
PS 2	4867	C18H38O10	46.4	Nonaethylene glycol	414.2470	415.2543	9.95
PS 3	none identified						
PS 4	10180	C20H24N2OS	42.2	1-[2-(diethylamino)ethylamino]-4-methylthioxanthen-9-one		379.1237	24.08
PP 1	12298194	C22H28N2O2	50.1	methyl (3S,4R)-4-anilino-3-methyl-1-(2-phenylethyl)piperidine-4-carboxylate	352.2162	353.2235	13.84
PP 1	16206038	C15H28O7	44.7	1-[2,3-bis(2-hydroxypropoxy)propoxy]propan-2-yl prop-2-enoate		343.1734	15.52
PP 1	117007	C9H14N2O2	41	4-(2-methoxyethoxy)benzene-1,3-diamine		387.1995	16.13
PP 1	119970	C14H31NO2	41.6	1-[2-hydroxypropyl(octyl)amino]propan-2-ol	245.2358	246.2430	18.85
PP 1	352309	C16H35NO2	50.2	Lauryldiethanolamine	273.2675	274.2747	22.18
PP 1	139595991	C21H32O8	42.3	1-[3-(2-carboxycyclohexanecarbonyl)oxy-2,2-dimethylpropoxy]carbonylcyclohexane-1-carboxylic acid	412.2107	435.1999	22.44
PP 1	48194	C22H27NO3	41.7	ethyl 4-morpholin-4-yl-2,2-diphenylbutanoate	353.2007	354.2079	22.51
PP 2	113595	C24H42O13	52.8	1-[3-[3-[2,3-bis(oxiran-2-ylmethoxy)propoxy]-2-hydroxypropoxy]-2-(oxiran-2-ylmethoxy)propoxy]-3-(oxiran-2-ylmethoxy)propan-2-ol	538.2632	561.2524	15.34

Sample	PubChem CID	Formula	Score	Description	Neutral mass (Da)	m/z	Retention time (min)
PP 2	66197	C10H16O5	43.2	diethyl 2-acetylbutanedioate	216.1002	455.1896	16.47
PET 1	5541	C9H14O6	41.8	Triacetin	218.0791	241.0684	10.76
PET 1[a]	53421640	C21H33N3O3	41.6	N-[3,5-bis(2,2-dimethylpropanoylamino)phenyl]-2,2-dimethylpropanamide	375.2520	376.2593	20.13
PVC 1	3024241	C9H14O4	49.9	1-[[5-(hydroxymethyl)furan-2-yl]methoxy]propan-2-ol		373.1869	17.80
PVC 1	53461848	C27H36O6S	44.4	ethyl 7-acetylsulfanyl-10,13-dimethyl-2',3-dioxospiro[2,6,7,8,9,11,12,14,15,16-decahydro-1H-cyclopenta[a]phenanthrene-17,5'-oxolane]-3'-carboxylate	471.2213		17.80
PVC 1	3024241	C9H14O4	47.1	1-[[5-(hydroxymethyl)furan-2-yl]methoxy]propan-2-ol		373.1865	19.16
PVC 1	19675	C24H30N2O2S	40	1-[10-[3-[4-(2-hydroxyethyl)piperidin-1-yl]propyl]phenothiazin-2-yl]ethanone	410.2047	843.3986	22.53
PVC 2[a]	81779	C24H51O10P	42.5	tris[2-(2-butoxyethoxy)ethyl] phosphate	530.3234	531.3307	25.61
PVC 3	12308635	C29H44O8	41.1	3-[(3S,5R,8R,9S,10S,13R,14S,17R)-14-hydroxy-10,13-dimethyl-3-[(2R,3R,4R,5R,6S)-3,4,5-trihydroxy-6-methyloxan-2-yl]oxy-1,2,3,4,5,6,7,8,9,11,12,15,16,17-tetradecahydrocyclopenta[a]phenanthren-17-yl]-2H-furan-5-one	520.3043	543.2935	23.68
PVC 3	81779	C24H51O10P	43.7	tris[2-(2-butoxyethoxy)ethyl] phosphate	530.3229	531.3302	25.61
PVC 3	11046239	C18H34O6	42.1	[(2R)-2-[(2R,3R,4S)-3,4-dihydroxyoxolan-2-yl]-2-hydroxyethyl] dodecanoate	346.2359	369.2251	25.91
PVC 4	6540	C18H39O7P	44.1	Tri(butoxyethyl)phosphate	398.2438	399.2510	25.56
PVC 4	6455034	C13H22O	41.8	2-methyl-4-(2,2,3-trimethylcyclopent-3-en-1-yl)butanal		227.2012	27.25
PVC 4	75403	C24H48O8	47.5	2-[2-[2-[2-[2-(2-hydroxyethoxy)ethoxy]ethoxy]ethoxy]ethoxy]ethyl dodecanoate	464.3335	487.3227	27.27
PUR 1	none identified						
PUR 2	none identified						
PUR 3	none identified						
PUR 4	22838325	C26H47NO6	42.3	(2-hydroxy-3-octadecanoyloxypropyl) (2S)-5-oxopyrrolidine-2-carboxylate	469.3383	492.3275	1.14
PLA 1	63216	C20H21N3O3	40.3	1-(2-morpholin-4-ylacetyl)-3-phenyl-2H-quinazolin-4-one		374.1459	26.29
PLA 2	none identified						
PLA 3	37907	C15H17ClN2O2	48.3	Climbazole	292.0986	293.1059	17.18
PLA 3	6454788	C10H16O4	43.6	6-but-3-enoxy-6-oxohexanoic acid	200.1048	423.1988	20.77
PLA 3	6454788	C10H16O4	46.4	6-but-3-enoxy-6-oxohexanoic acid	200.1056	401.2186	20.77
PLA 4	20836181	C19H37NO	47.4	(Z)-N-methyloctadec-9-enamide		296.2939	30.10
PLA 4	26396	C10H18O2	43	6-ethenyl-2,2,6-trimethyloxan-3-ol		341.2676	30.16

Note: [a] listed in the CPPdb as associated with plastic packaging.

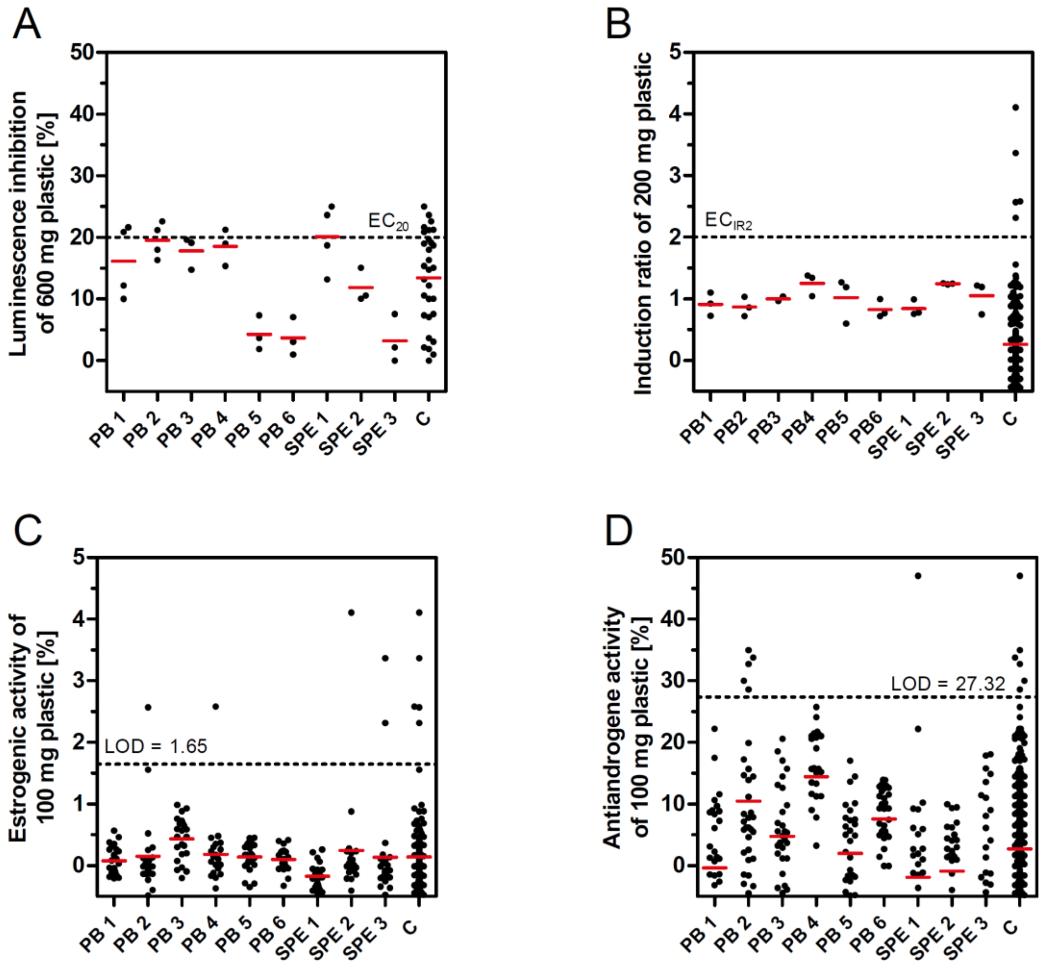

Figure S1. Effects of the procedural (PB) and SPE blanks (SPE) of the experiments with migrates (SPE extracted) as well as their pooled values (= controls; C) in the *in vitro* bioassays. PBs, SPEs, and C do not induce effects in the Microtox assay (*A*, *n* = 3–4 in duplicates), the AREc32 assay (*B*, *n* = 2–5 in duplicates), the yeast-based reporter-gene assays for estrogenic activity (*C*, *n* = 24 from three independent experiment) and antiandrogenic activity (*D*, *n* = 24–80 from ≥ 3 independent experiment). The results are presented as means (line) of *n* (dots). The corresponding cut-off levels of each assay (effect concentrations (EC) and limits of detection (LOD)) were not exceeded.

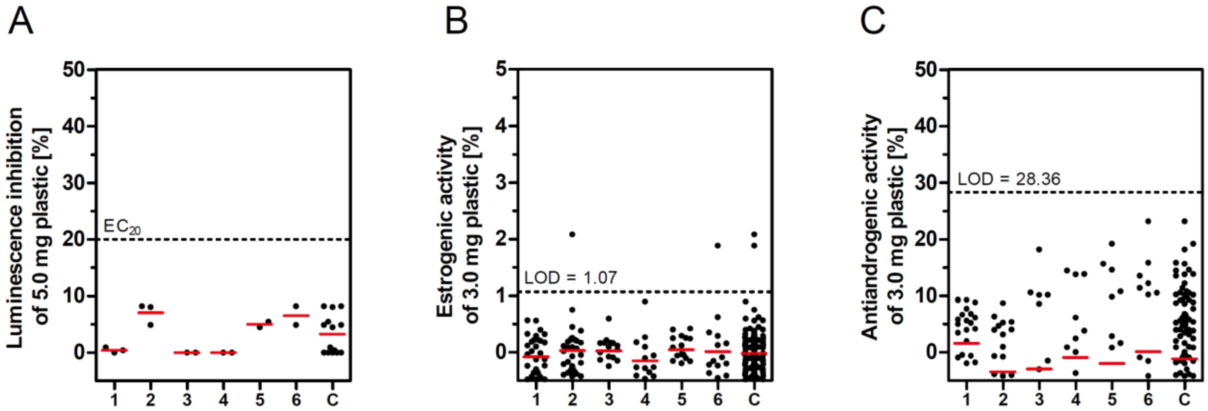

Figure S2. Effects of procedural blanks 1–6 of the experiments with aqueous migrates as well as their pooled values (= controls; C) in the *in vitro* bioassays. Procedural blanks and C do not induce effects in the Microtox assay (*A*, *n* = 2–3 in duplicates), the yeast-based reporter-gene assays for estrogenic activity (*B*, *n* = 16–32 from three independent experiment) and antiandrogenic activity (*C*, *n* = 16–24 from ≥ 3 independent experiment). The results are presented as means (line) of *n* (dots). The corresponding cut-off levels of each assay (effect concentrations (EC) and limits of detection (LOD)) were not exceeded.

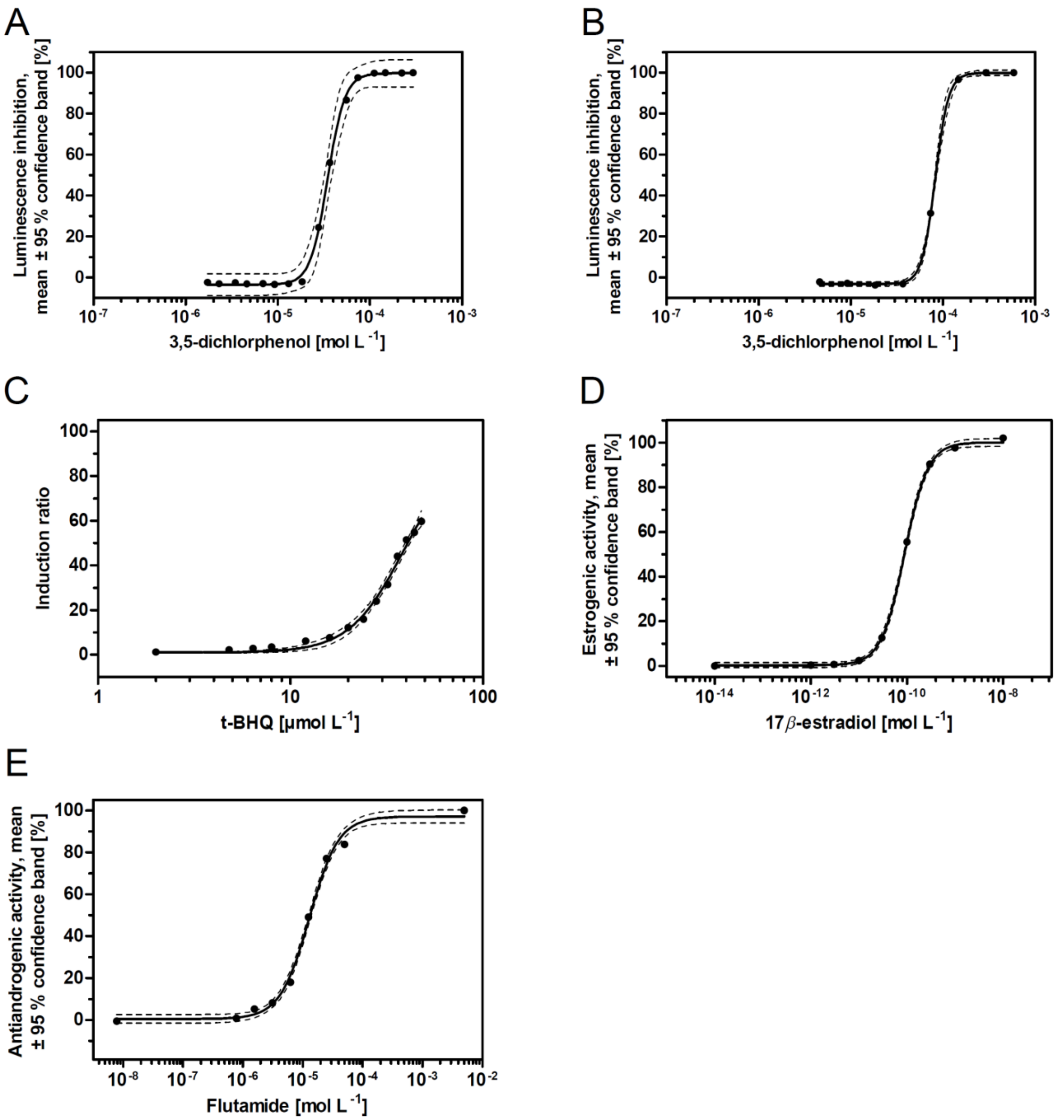

Figure S3. Dose-response relationship of the reference compounds in the bioassays. Microtox (*n* = 3), measured in different concentrations for migrates (*A*) and aqueous migrates (*B*), AREc32 assay (*C*, *n* = 8), YES (*D*, *n* = 32), and YAAS (*E*, *n* = 32). In addition to the full dose-response curve of *tert*-butylhydroquinone (t-BHQ) analyzed in the AREc32 assay once, a serial dilution (1:2) of t-BHQ (10^{-5} M) was included on every 96-well plate in order to ensure a comparable sensitivity of the cells of different passages.

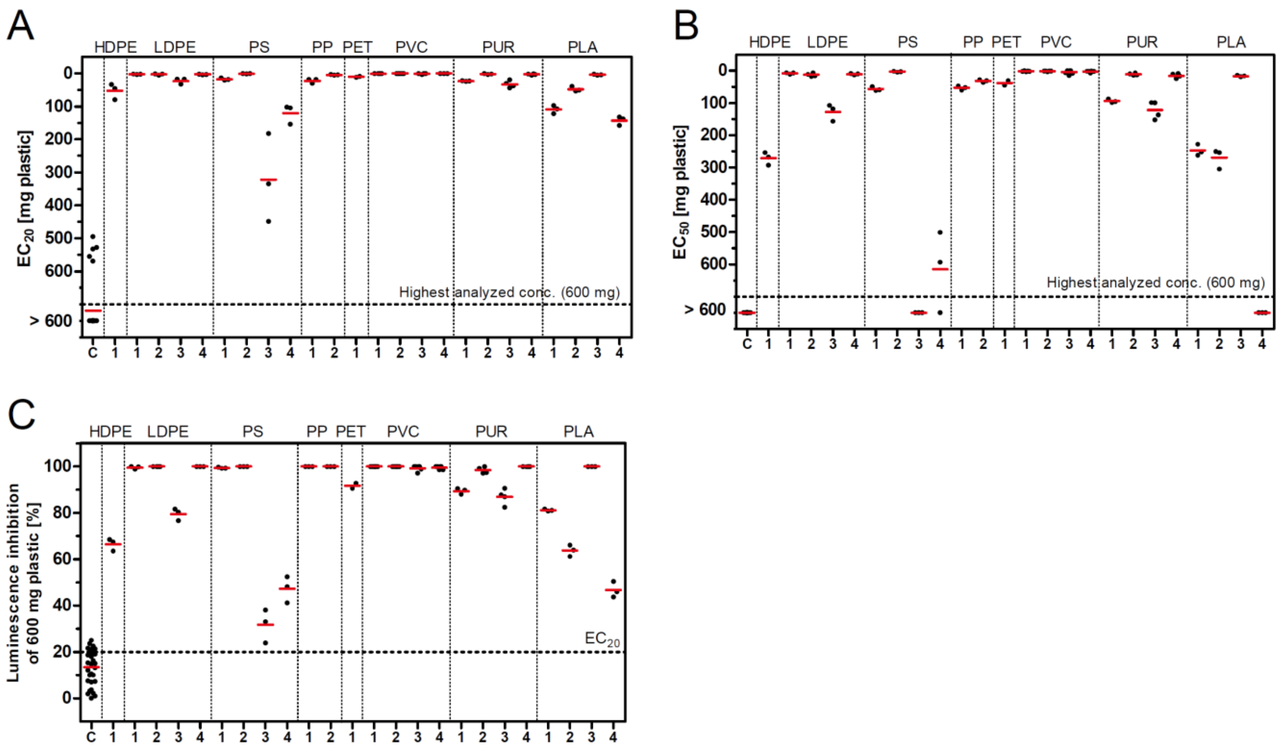

Figure S4. Baseline toxicity of plastic migrates in the Microtox assay presented as means (lines) EC_{20} (*A*) and EC_{50} (*B*) baseline toxicity as well as luminescence inhibition (*C*). >600 indicates that the migrate of 600 mg plastic well^{-1} did not inhibit the bioluminescence by >20 or 50 %, respectively.

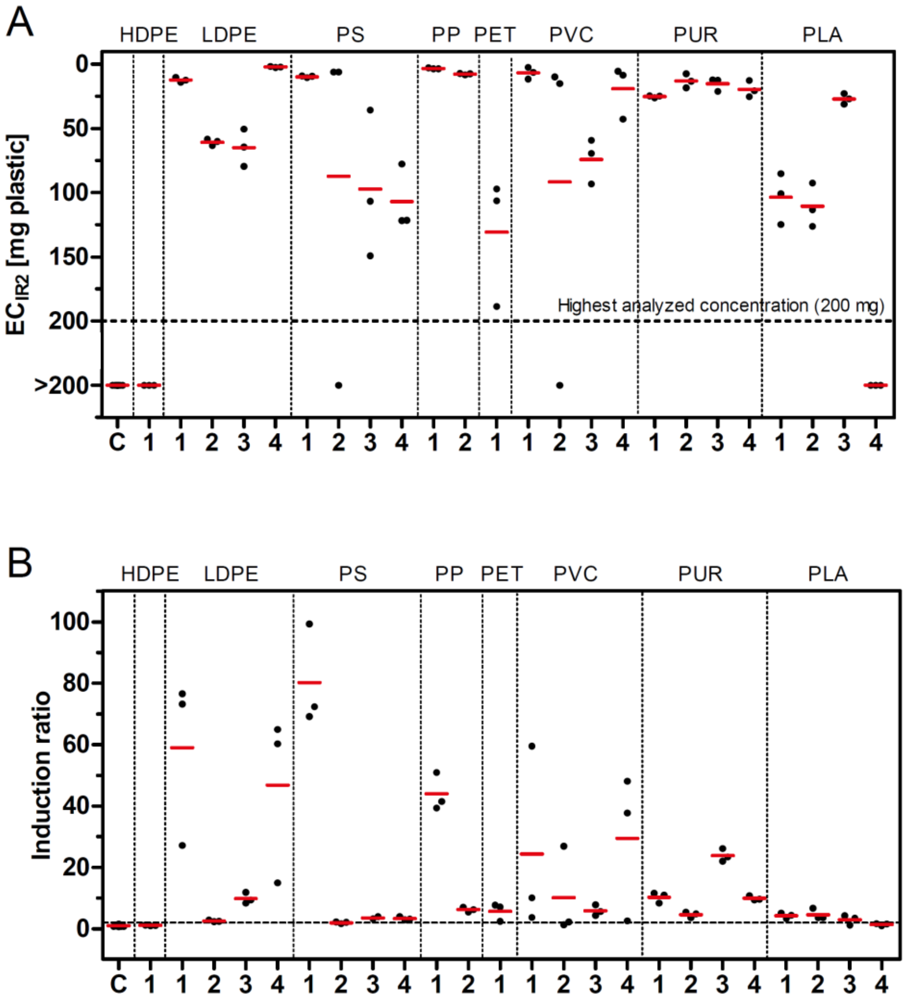

Figure S5. Oxidative stress response induced by migrates in the AREc32 assay as mean (lines) EC_{IR2} (*A*) and induction ratios of the highest measured non-cytotoxic concentrations (*B*, see Table S5) >200 indicates that migrates of 200 mg plastic well^{-1} (highest analyzed concentration) did not exceed an induction ratio of 2.

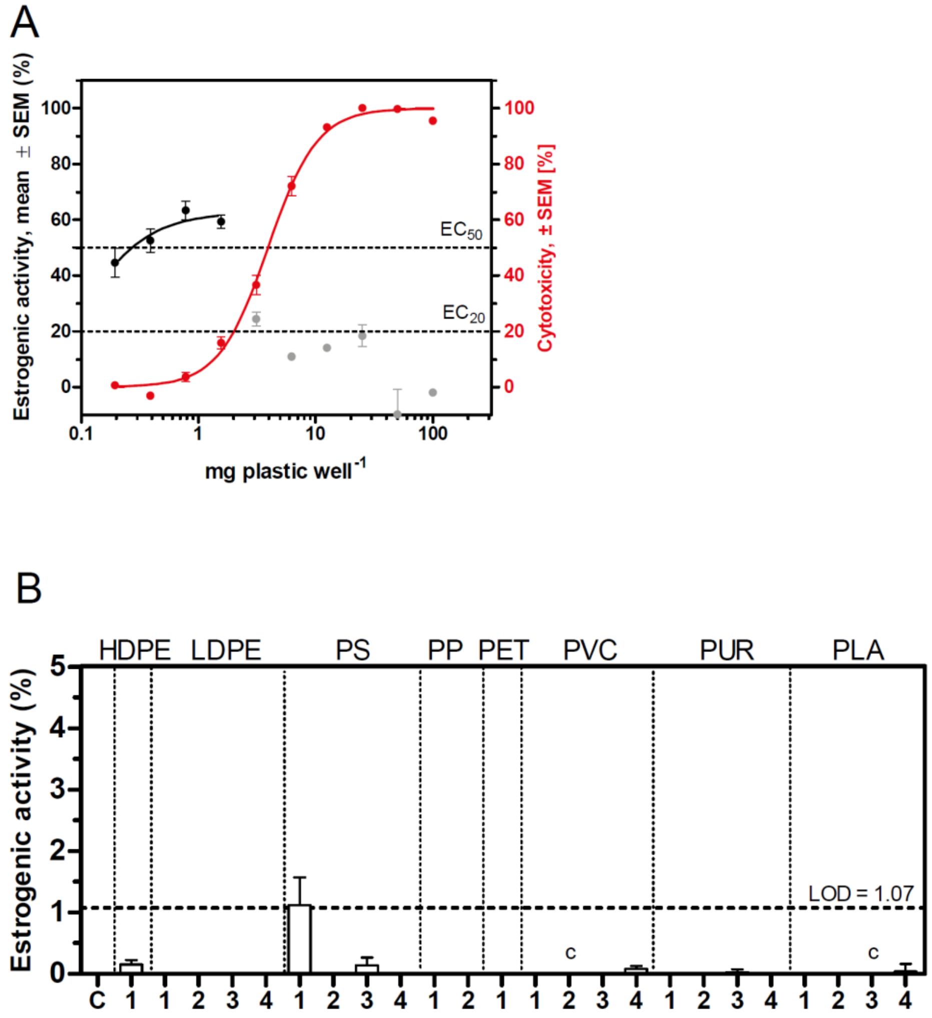

Figure S6. Relative estrogenic activities in the Yeast Estrogen Screen (*n* = 16–24). (*A*) Activity data of non-cytotoxic (black) and cytotoxic concentrations (c, grey) of PVC 2, the only migrate with estrogenic activity (mean effects > LOD, Table S6). (*B*) Relative receptor activation of aqueous migrates from 3.00 mg plastic well^{-1}.

A

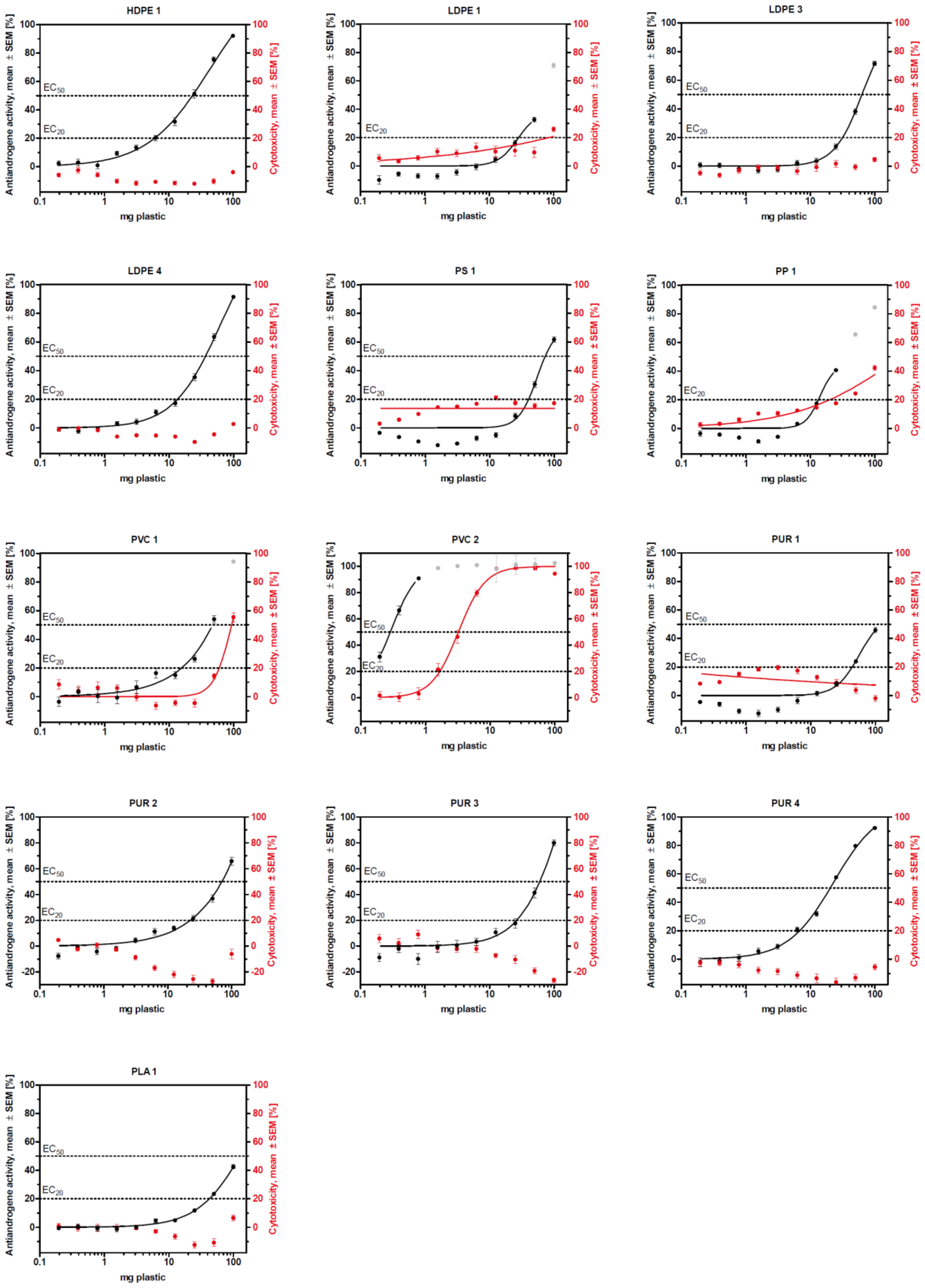
HDPE 1
LDPE 1
LDPE 3
LDPE 4
PS 1
PP 1
PVC 1
PVC 2
PUR 1
PUR 2
PUR 3
PUR 4
PLA 1
Antiandrogene activity, mean ± SEM [%]
Cytotoxicity, mean ± SEM [%]
mg plastic
EC_{50}
EC_{20}

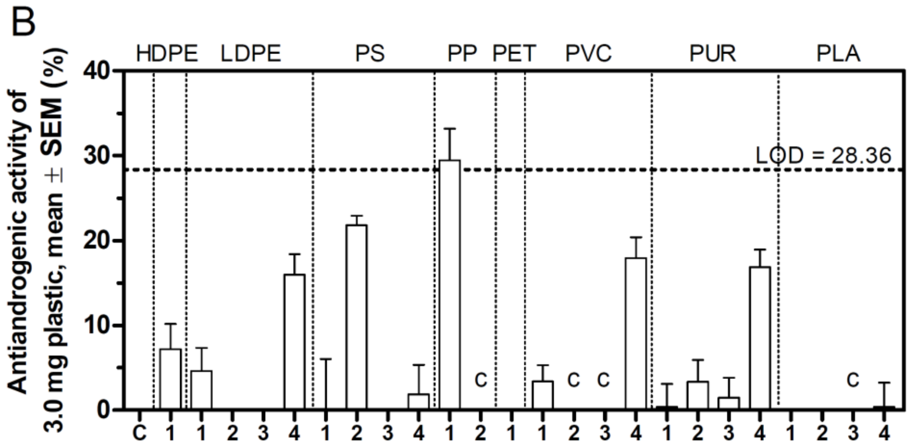

Figure S7. Relative antiandrogenic activities in the Yeast Antiandrogen Screen (n = 16–32). (*A*) Activity data of migrates which were antiandrogenic (mean effects > LOD) at 100 mg plastic well^{-1} presented with their cytotoxic (c) concentrations (grey) and non-cytotoxicity concentrations (black). (*B*) Aqueous migrates from 3.00 mg plastic well^{-1}.

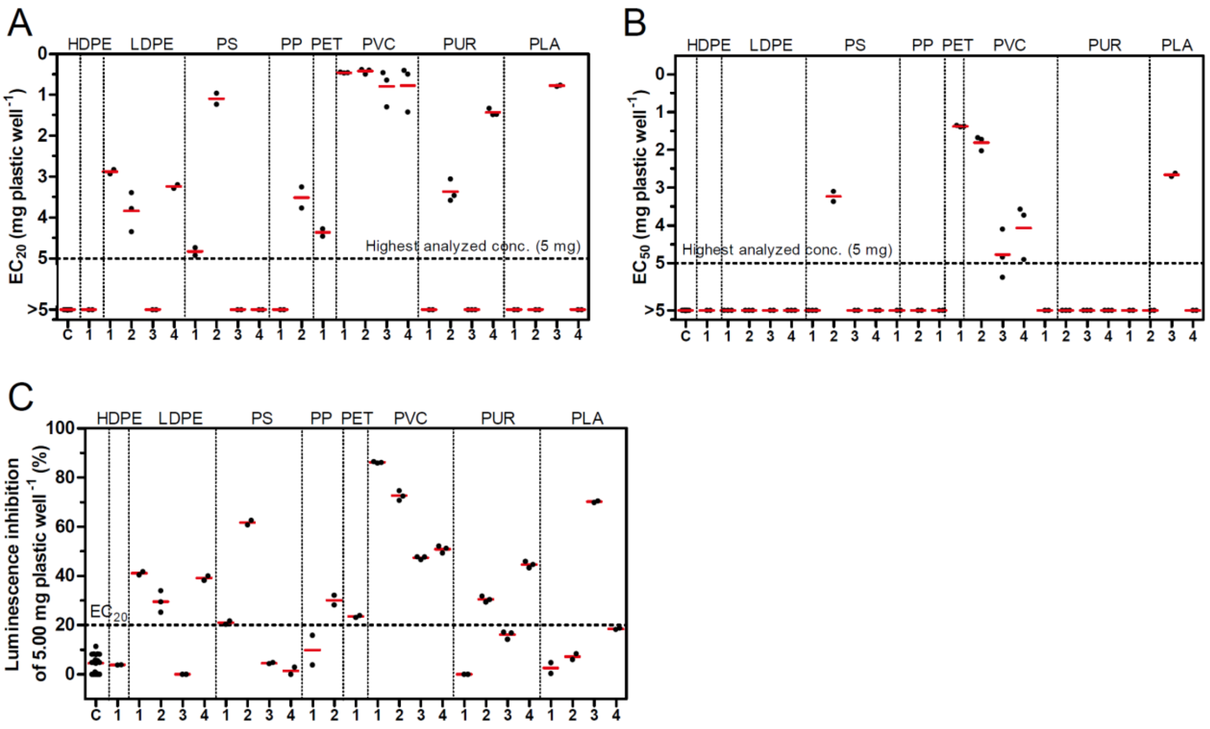

Figure S8. Baseline toxicity of aqueous plastic migrates in the Microtox assay presented as means (lines) EC$_{20}$ (*A*) and EC$_{50}$ (*B*) baseline toxicity as well as luminescence inhibition (*C*) from two to three independent experiments (dots). >5 indicates that migrates of 5.00 mg plastic well^{-1} did not inhibit the bioluminescence by > 20% or 50 %, respectively.

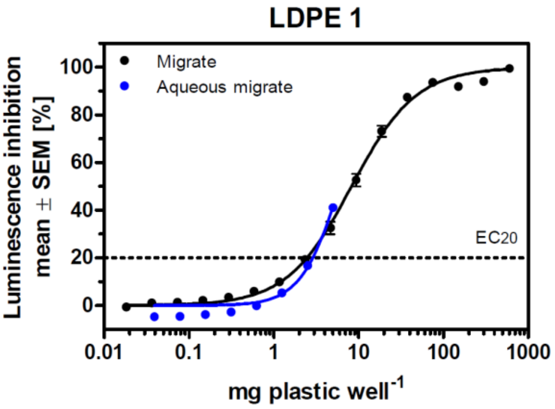
LDPE 1
Migrate
Aqueous migrate
EC20
Luminescence inhibition mean ± SEM [%]
mg plastic well⁻¹

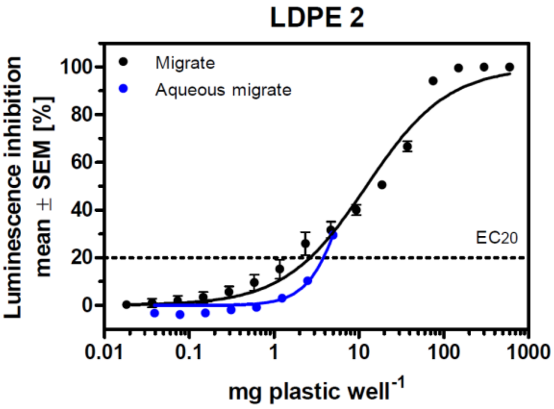
LDPE 2
Migrate
Aqueous migrate
EC20
Luminescence inhibition mean ± SEM [%]
mg plastic well⁻¹

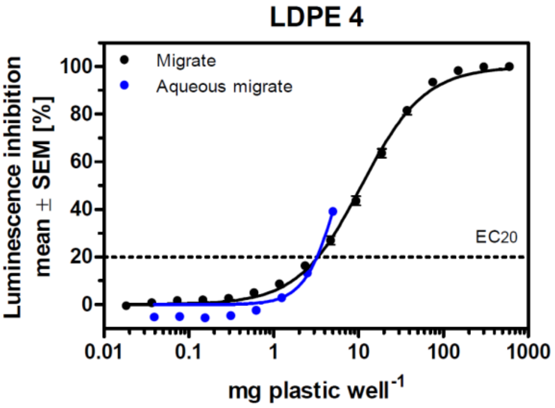
LDPE 4
Migrate
Aqueous migrate
EC20
Luminescence inhibition mean ± SEM [%]
mg plastic well⁻¹

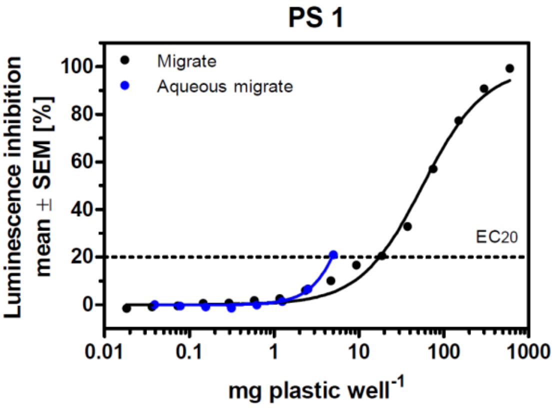
PS 1
Migrate
Aqueous migrate
EC20
Luminescence inhibition mean ± SEM [%]
mg plastic well⁻¹

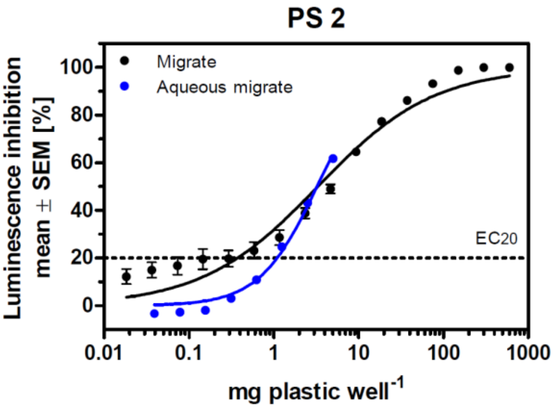
PS 2
Migrate
Aqueous migrate
EC20
Luminescence inhibition mean ± SEM [%]
mg plastic well⁻¹

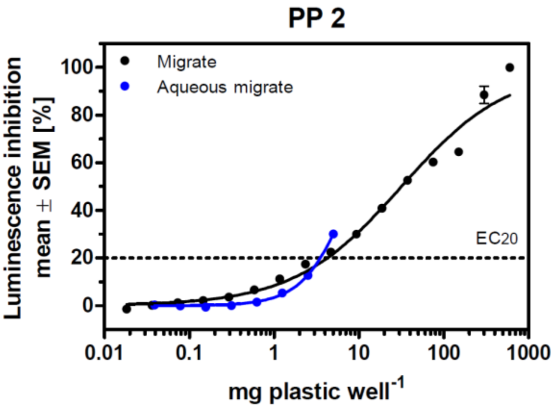
PP 2
Migrate
Aqueous migrate
EC20
Luminescence inhibition mean ± SEM [%]
mg plastic well⁻¹

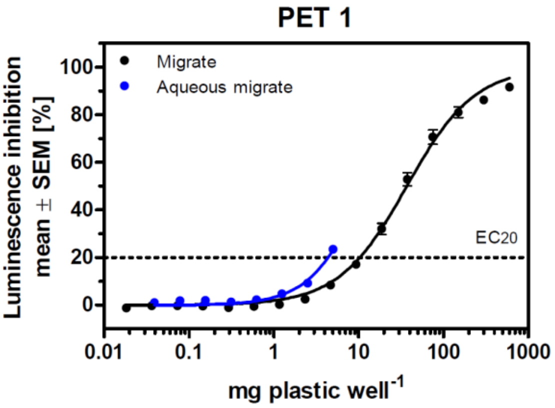
PET 1
Migrate
Aqueous migrate
EC20
Luminescence inhibition mean ± SEM [%]
mg plastic well⁻¹

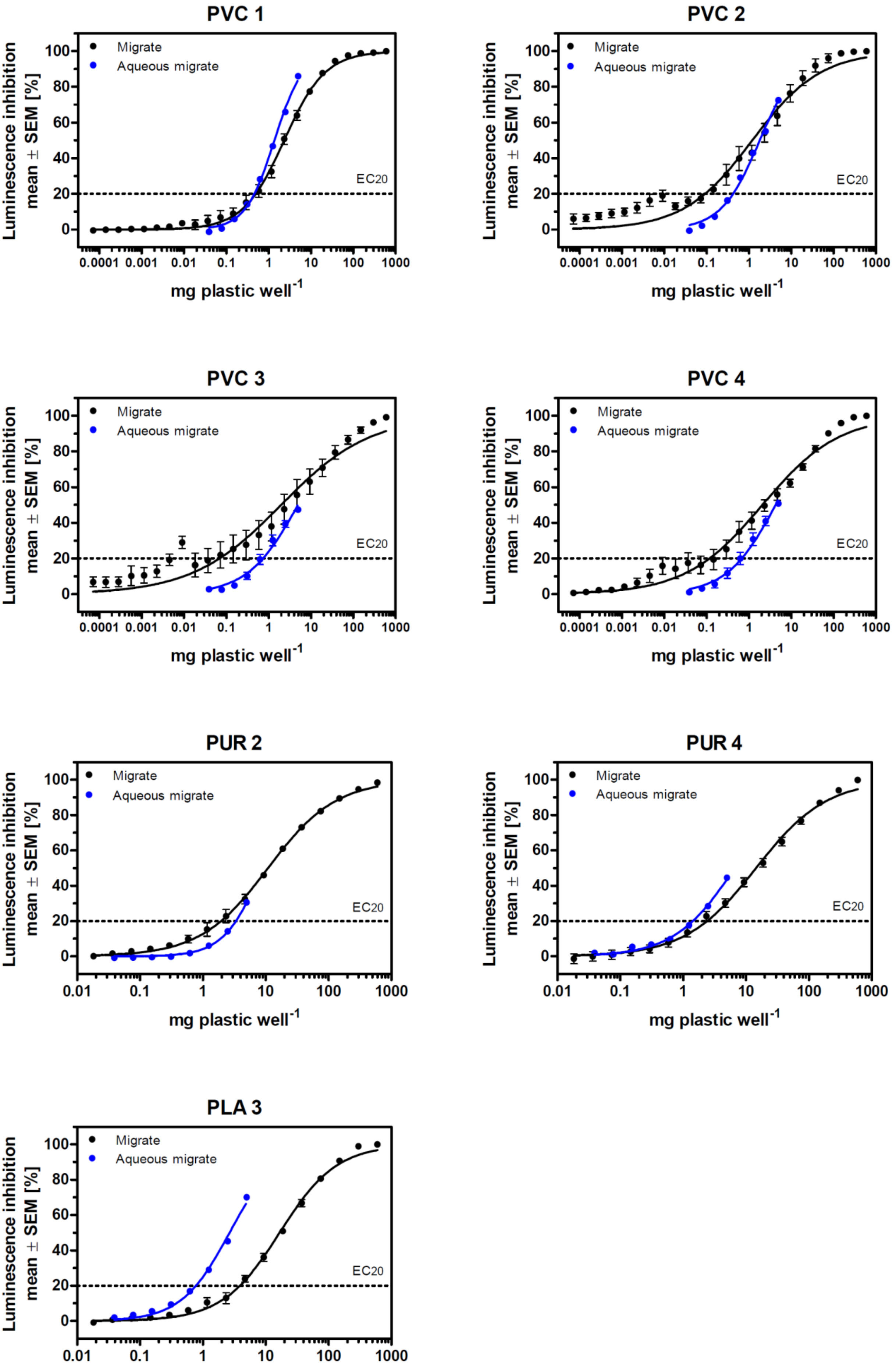
PVC 1
PVC 2
PVC 3
PVC 4
PUR 2
PUR 4
PLA 3
Migrate
Aqueous migrate
EC20
Luminescence inhibition mean ± SEM [%]
mg plastic well^{-1}
0
20
40
60
80
100
0.0001
0.001
0.01
0.1
1
10
100
1000

Figure S9. Concentration-response relationship for baseline toxicity of migrates (with SPE) and aqueous migrates (without SPE). Only samples for which both, migrates and aqueous migrates, induced luminescence inhibition ≥ 20 % at measured concentrations are shown. Data is presented as mean ± standard error of the mean (SEM). EC_{20}, effect concentration inducing 20 % baseline toxicity.

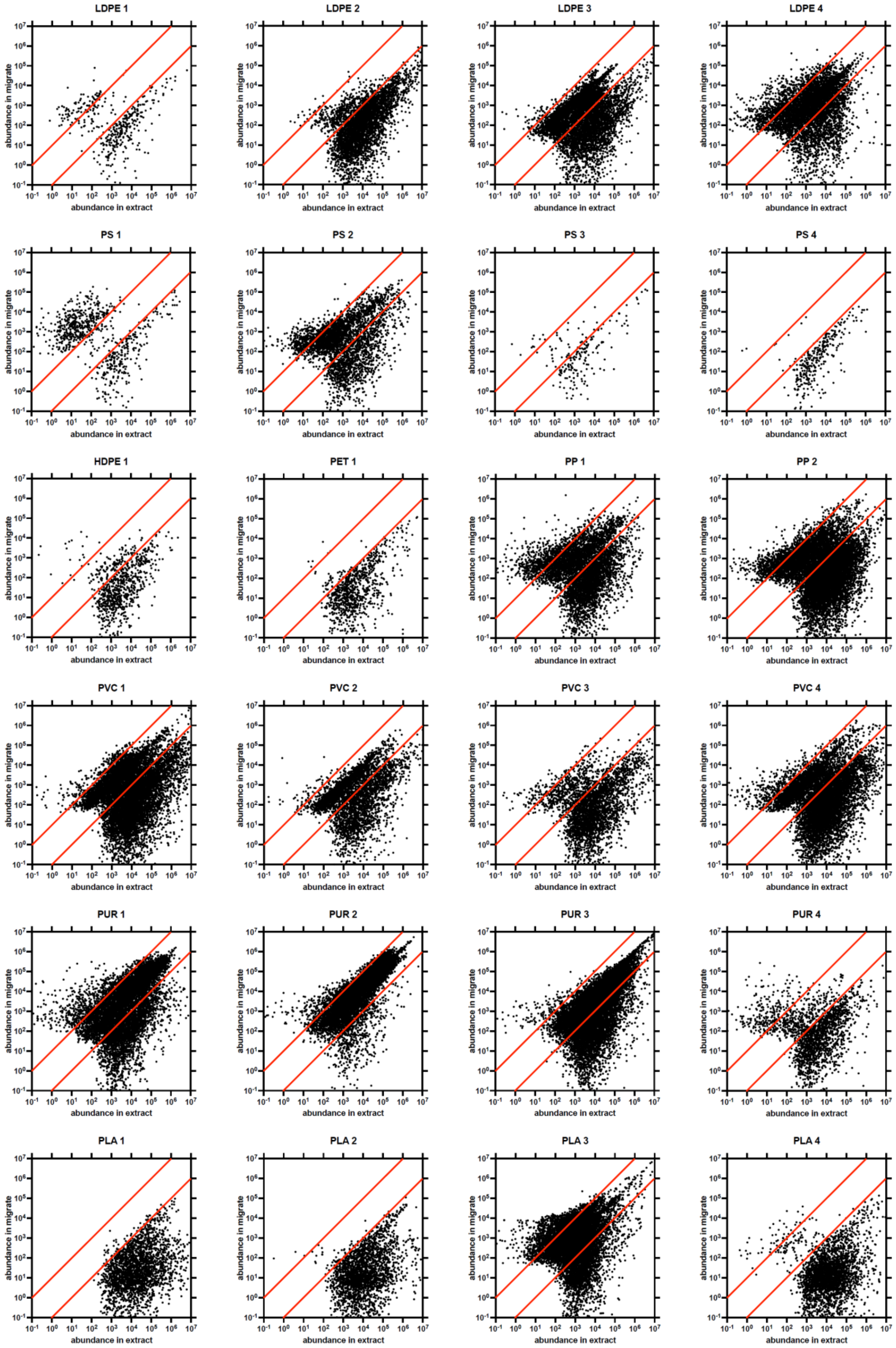

Figure S10. Comparison of the abundance of chemical features detected in the extracts and migrates of each plastic product. Red lines highlight the band of an abundance ratio of 0.1–10.

Supplementary references

(1) European Commission (EC). Regulation (EU) No 10/2011 of 14 January 2011 on plastic materials and articles intended to come into contact with food, 2014.

(2) Groh, K., Schymanski, E. S48 | CCPDBLISTA | Database of Chemicals likely (List A) associated with Plastic Packaging (CPPdb) (Version NORMAN-SLE-S48.0.1.0) [Data set]. Zenodo, 2019.

(3) Groh, K., Schymanski, E. S49 | CCPDBLISTB | Database of Chemicals possibly (List B) associated with Plastic Packaging (CPPdb) (Version NORMAN-SLE-S49.0.1.0) [Data set]. Zenodo, 2019.

(4) Alygizakis, N., Slobodnik, J. S32 | REACH2017 | >68,600 REACH Chemicals (Version NORMAN-SLE-S32.0.1.4) [Data set]. Zenodo, 2018.
